# Morphological Characterization, Essential Oil Composition and Antibacterial Activity of Some Cultivars from the Genera *Monarda*, *Satureja*, *Thymus*, *Ocimum* and *Hyssopus* Grown in the Central Forest-Steppe Zone of Ukraine

**DOI:** 10.3390/molecules31213946

**Published:** 2026-11-02

**Authors:** Nataliia Hudz, Oksana Yezerska, Serhii Svydenko, Tetiana Rumynska, Andrii Lozynskyi, Mariia Shanaida, Liudas Ivanauskas

**Affiliations:** 1 Institute of Chemistry, University of Opole, 45-052 Opole, Poland; 2 Department of Drug Technology and Pharmacognosy, Danylo Halytsky Lviv National Medical University, Pekarska Street 69, 79010 Lviv, Ukraine; yezerska_oksana@lnmu.edu.ua; 3 Institute of Climate-Smart Agriculture of the NAAS of Ukraine, Khlibodarske, 67667 Odesa, Ukraine; svydenkosv@gmail.com; 4 Department of Microbiology, Danylo Halytsky Lviv National Medical University, Pekarska Street 69, 79010 Lviv, Ukraine; tanityshka.r@ukr.net; 5 Department of Pharmaceutical, Organic and Bioorganic Chemistry, Danylo Halytsky Lviv National Medical University, Pekarska Street 69, 79010 Lviv, Ukraine; lozynskyiandrii@gmail.com; 6 Department of Pharmacognosy and Medical Botany, I. Horbachevsky Ternopil National Medical University, 46001 Ternopil, Ukraine; shanayda@tdmu.edu.ua; 7 Department of Analytical and Toxicological Chemistry, Lithuanian University of Health Sciences, A. Mickeviciaus g. 9, LT-44307 Kaunas, Lithuania; liudas.ivanauskas@lsmu.lt

**Keywords:** Lamiaceae, *Monarda*, *Satureja*, *Thymus vulgaris*, *Ocimum basilicum*, *Hyssopus officinalis*, essential oil, antibacterial activity, GC-MS, chemotype, monoterpenoids

## Abstract

Representatives of five genera of the Lamiaceae family, such as *Thymus*, *Satureja*, *Monarda*, *Ocimum,* and *Hyssopus*, are especially appreciated for their valuable decorative features and the beneficial biological properties of their essential oils and extracts. In this study, the key morphological features of eight new cultivars *(Monarda didyma* L., *Monarda bradburiana* L. C. Beck, *Satureja hortensis* L., *Satureja coerulea* L., *Thymus vulgaris* L., *Ocimum basilicum* var. citriodorum L., and *Hyssopus officinalis* L.), cultivated firstly under the conditions of the central forest-steppe of Ukraine, as well as the chemical composition and antibacterial activity of their essential oils, were studied. The chemical composition of the essential oils obtained from the fresh aerial parts of the studied plants via hydrodistillation was evaluated by GC-MS. In addition, the screening tests of antibacterial activity against *Staphylococcus aureus* (ATCC 25923), *Staphylococcus epidermidis* (ATCC 12228), *Pseudomonas aeruginosa* (ATCC 27853), *Klebsiella pneumoniae* (clinical isolate), and *Escherichia coli* (ATCC 25922) were performed using the disk diffusion method. Two essential oils, *T. vulgaris* and *M. didyma*, were rich in thymol (49.9% and 58.5%, respectively), and four essential oils (*M. didyma*, *M. bradburiana*, *S. hortensis*, and *S. coerulea*) were rich in carvacrol (49.4–69.9%). The essential oil of *O. basilicum* contained linalool, citral, and neral (38.7%, 12.6%, and 9.4%), whereas the essential oil of *H. officinalis* contained pinanone (51.3%) and pinocarvone (18.5%). All the tested essential oils exhibited strong antibacterial activity against *S. aureus* and *S. epidermidis*. The essential oils of *M. didyma*, *M. bradburiana*, *S. hortensis*, *S. coerulea*, *T. vulgaris*, and *O. basilicum* had strong antibacterial activity against *E. coli* as well. Our findings demonstrated that the studied essential oils are rich in characteristic terpenes and terpenoids, which are likely to induce their antimicrobial activity. The revealed morphological features, chemical composition and antibacterial activity of the essential oils highlight the need for further studies to assess the repeatability of results for these cultivars bred across other years on the same plots. Additionally, the studied plants have great potential for further research on the development of medicinal and cosmetic products with various biological properties.

## 1. Introduction

Essential oil-bearing plants are a valuable source of various bioactive substances, including, first of all, essential oils that are obtained through hydrodistillation or steam distillation [1,2,3,4]. Essential oils found in the Lamiaceae species are synthesized and accumulated in glandular trichomes of the epidermis [3].

Nowadays, there is growing interest in essential oils due to their antimicrobial, antioxidant, and anti-inflammatory activities [1,2,5,6]. The development of antibiotic resistance and undesirable side effects of some antimicrobial drug substances have caused the search for new antimicrobial substances of natural origin [1,2,6,7,8].

Myriad studies demonstrate that not only do various essential oils show antimicrobial activity, but other valuable biological effects [5,8,9]. For example, Côté et al. [9] revealed that the essential oil of *Monarda didyma* L. had metabolic activity. Broilers fed with the essential oil diet were significantly heavier compared to the control broilers fed with antibiotics [9]. Therefore, essential oils could substitute antibiotic growth promoters, which are prohibited in certain countries. For this reason, producers are forced to remove antibiotic growth promoters as feed additives for poultry from the perspective of the protection of human health. Essential oil of *M. didyma* could be an alternative to synthetic fungicides, which are currently used in the control of the devastating fungal pathogen *Botrytis cinerea* Pers. that causes grey mold disease of strawberry [10].

The *Thymus vulgaris* L. essential oil can be used as a natural supplement to extend the shelf life of food [7], for use in mouth rinses, toothpastes, or for aromatherapy [11]. It was observed that the essential oil of *M. didyma* had anti-inflammatory activity because of an overexpression of miR-146a, down-regulation of IRAK-1, and reduction in the pro-inflammatory cytokine IL-6 production [12,13]. *M. fistulosa* and *M. bradburiana* L. C. Beck essential oils had good repellent activity against the yellow fever mosquito *Aedes aegypti* with minimum effective dosages of 0.078 ± 0.027 and 0.055 ± 0.036 mg/cm^2^, respectively, compared to the reference standard N,N-diethyl-3-methylbenzamide (0.039 ± 0.014 mg/cm^2^) [14]. Therefore, essential oils could be used for the development of topical medicinal products with antimicrobial and anti-inflammatory activities and cosmetic products as well. However, for pharmaceutical purposes, essential oils should have a more or less constant chemical composition. In addition, some essential oils are used for aromatherapy [15,16].

The antimicrobial activity of essential oils of the Lamiaceae family is mainly attributed to thymol and carvacrol, eugenol, and *p*-cymene. The first two biologically active substances damage the membrane integrity with changes in pH homeostasis and the equilibrium of inorganic ions, while *p*-cymene, not having antimicrobial activity, increases the antimicrobial activity of thymol or carvacrol [8,9,11,17]. Moreover, thymol and carvacrol are believed to be effective against Gram-negative bacteria as well. These compounds act on the outer membrane and increase the permeability of the cytoplasmic membrane causing the release of lipopolysaccharides, intracellular ATP and potassium ions, and depolarizing the cytoplasmic membrane. In addition, hydroxyl groups form hydrogen bonds with enzymes, inactivating them [9,18]. γ-Terpinene also demonstrates antimicrobial activity. Furthermore, numerous studies show that essential oils have stronger antimicrobial activity compared to their principal components. This fact suggests synergistic antimicrobial effects of the major and minor constituents [17,19].

Chen et al. [6] supposed that the essential oil of *M. didyma* damages the cell membrane, causing the leakage of macromolecules, especially intracellular soluble proteins. It is worth mentioning that according to Krause et al. [20] thymol and carvacrol are present only in few genera of the Lamiaceae family (*Thymus*, *Satureja*, *Origanum*, and *Thymbra*), Apiaceae (*Trachyspermum*), and Verbenaceae (*Lippia*). Additionally, the *Monarda* genus can be also referred to the genus that contains thymol and carvacrol as well [10,12,21].

It is thought that essential oils containing antimicrobial, antifungal, insecticidal and anti-germination compounds against the seeds of several weeds are considered to be a chemical defense for plants to adapt and survive in unfavorable environmental conditions [3,21].

The Lamiaceae family is believed to be the most valuable, considering the rich chemical composition of some members [1,9,11]. Genera *Satureja*, *Monarda*, *Thymus*, *Mentha*, *Origanum*, *Lavandula*, *Hyssopus*, *Thymbra*, and *Coridothymus* appeal scientists’ attention in different areas of science (biology, chemistry, agriculture, pharmacy, medicine and cosmetology) [3,8,10,11,21,22,23,24,25,26,27,28]. Moreover, some species are cultivated for many reasons, especially for obtaining essential oils, for their noticeable ornamental value, and for use in culinary applications [3,4,21,28,29].

The genus *Monarda* embraces 20 species native to North America and is divided into two distinct subgenera, *Monarda* and *Cheilyctis* [3,5,19]. The subgenus *Monarda* embraces *M. didyma* L., *M. fistulosa* L., *M. bradburiana* L. C. Beck, *M.* × media Willd., *M. clinopodia* L., whereas subgenus *Cheilyctis* includes *M. citriodora* Cerv. ex-Lag., *M. punctata* L., *M. clinopodioides* A. Gray, and *M. pectinata* Nutt [19]. This genus is rich in phenolic substances of various classes, including flavonoids and phenolic acids, and monoterpenoids [2,3,5,12,19,21,28].

Different chemotypes of *Monarda* essential oils have been described and characterized by major constituents such as thymol, carvacrol, borneol, and geraniol [5,12]. In general, essential oils from aerial parts of *Monarda* plants contain main components such as thymol and/or carvacrol [12,19,21]. *M. didyma* L. and *M. fistulosa* L. were first imported to Ukraine in the middle of the 20th century. *M. fistulosa* has a broader distribution compared to *M. didyma* because of higher yields of essential oil and much better aroma [30].

*M. didyma* (Oswego tea, Bee Balm, Golden Balm, and Wild Bergamot) is an aromatic perennial plant with a broad range of culinary, medicinal and cosmetic applications [1,28]. The leaves have a pleasant taste and are used to prepare a digestive and expectorant tea, whereas the plant’s resins are used to soothe bee stings. The plant is aromatic due to the presence of glandular trichomes containing essential oil (capitate glandular trichomes, short three-celled trichomes and peltate trichomes) [28]. *M. didyma* is rich in polyphenols, including flavonoids. Among the flavonoids are glycosides of apigenin, kaempferol, and diosmin [28].

Eight chemotypes of *M. didyma* essential oil were identified, containing such dominant components as cymene, γ-terpinene, linalool, thymol methyl ether, thymol, carvacrol, borneol, and geraniol [1]. The principal compounds which can represent the chemotype could be thymol, linalool, carvacrol, geraniol or borneol [12].

*M. didyma* flowers are included in the group of edible flowers for human consumption [12,28]. In 2018, *M. didyma* flowering tops were included in Annex 1 of the Belfrit List of botanicals allowed in food supplements [28,31].

*M. bradburiana*, the eastern beebalm grows naturally in southcentral North America (southern Illinois, Missouri, Arkansas, Kentucky, Tennessee) [32]. It seems that it is an under investigated species [19]. This species is rich in rosmarinic acid (1.24 g/100 g in leaves), linarin (1.78 g/100 g) [19], and monoterpenoids [32].

*Satureja* is one of the biggest genera in the Lamiaceae family. This genus is distributed in various regions of the world. However, most of the species are found in the Mediterranean region and surrounding areas [3,26,33]. Twelve *Satureja* species are distributed across Europe [3,33].

It is worth noting that there are difficulties in the delimitation of *Satureja* species as they can easily hybridize within the same population and between different *Satureja* species. As a result, there is a high degree of morphological variability and overlapping characteristics within and among different species [3]. This fact can induce variability in the chemical composition of the essential oils as well [8,26]. Moreover, this genus has specific micromorphological features, namely, the epidermis of some plant parts has non-glandular trichomes. For instance, the leaves and calyx of *S. coerulea* Janka have non-glandular and glandular trichomes on the surfaces of both epidermises, while the stem has only unicellular or multicellular unbranched non-glandular trichomes [3,33].

The stems, leaves, flowers and seeds of a lot of *Satureja* species are used for the traditional treatment of different conditions such as diarrhoea, nausea, muscle aches, and infectious diseases [26]. Leaves, fruits, the top part with flowers, seeds and essential oil of *S. montana* L., and leaves, herb with flowers and essential oil of *S. hortensis* are in the Belfrit List of botanicals allowed in food supplements as well [31]. *S. montana* and *S. hortensis* are known for their medicinal properties and significant potential in the fields of aromatherapy, culinary, cosmetology, and nutrition [8,26].

The genus *Satureja* is rich in polyphenols. Shanaida et al. [2] identified rosmarinic acid (13.2 g/kg), chlorogenic acid (1.0 g/kg), glycosides of flavones (luteolin-7-O-glucoside (7.5 g/kg) and apigenin-7-O-glucoside (3.5 g/kg)), various flavonols (rutin (237.8 mg/kg), isoquercitrin (quercetin-3-glucoside) (117.3 mg/kg), and astragalin (kaempferol-3-glucoside) (123.2 mg/kg)) in the herb of *S. coerulea* of Ukrainian origin.

The genus *Thymus* embraces about 400 species of perennial aromatic, evergreen or semi-evergreen herbaceous plants with a plentiful number of subspecies, varieties, subvarieties, and forms. *T. vulgaris* (thyme) is widely used in folk medicine for its expectorant, antitussive, antispasmodic, anthelmintic, carminative and diuretic properties. There are six known chemotypes, namely, carvacrol, geraniol, γ-terpineol, linalool, thymol and trans-thujan-4-ol/terpinen-4-ol [7,11,17,20]. The essential oil is clear and light yellow with a pleasant odor [11].

Species of the genus Basil (*Ocimum*) are pleasant-smelling perennial shrubs which grow in several regions all over the world [29]. Traditionally, sweet basil is a popular culinary herb [29], and it is used as a medicinal plant in the treatment of headaches, coughs, diarrhea, constipation, warts, worms, and kidney malfunctions. The chemical composition and, accordingly, aromatic features of basil depend on genotype, chemotype, location of growth, agronomic practices and environmental conditions [18,29]. Essential oil of sweet basil consists of monoterpenes and phenylpropanoids. Among the major constituents are linalool, methyl chavicol, citral and 1,8-cineole, camphor, thymol, methyl cinnamate, eugenol, methyl eugenol, methyl isoeugenol, elemicine, methyl cinnamate, etc. There are four described chemotypes of basil: (1) methyl chavicol-rich, (2) linalool-rich, (3) methyl eugenol-rich, (4) methyl cinnamate-rich, and also numerous subtypes [18].

There are considerable variations in the contents of the principal components within this species. It was found that the main constituents of the *O. basilicum* essential oil are produced by two different biochemical pathways, the phenylpropanoids (methyl chavicol, eugenol, methyl eugenol and methyl cinnamate) by the shikimic acid pathway, and the terpenes (linalool and geraniol) by the mevalonic acid pathway [29].

The species within the genus *Hyssopus* L. are commonly found in the Eastern Mediterranean, Central Asia, and Mongolia [34]. According to the contemporary classifications, the genus *Hyssopus* comprises from seven to fifteen species of plants [34,35,36]. *H. officinalis* is a particularly important species that is widely used as an herbal remedy for treating various conditions, including colds, fungal infections, and spasmodic issues. Its bioactive components, especially essential oils, are also utilized for their aromatic properties in the food and cosmetic industries. Hyssop essential oil is primarily composed of oxygenated monoterpenoids of different structure [35,36]. According to Atazhanova et al. [35], there exist some chemotypes of *H. officinalis* (pinocamphonic, linalool, and thymolic). Moreover, there are some correlations between the color of flowers (white, blue and pink) and composition of essential oil.

As stated by Fraternale et al. [12], Özcan and Chalchat [29], and Nurzynska-Wierdak [36], the chemical composition of essential oil can be quite different from what has already been described for the same species. It is worth mentioning that the yield and chemical composition of essential oils depend on many factors. Among them are species, genetic features, geographical conditions, chemotype, environmental factors (soil, humidity, sunshine, length of the day), state of development, harvest period, cultivation techniques, different drying methods, distillation techniques, plant part, etc. [4,12,16,17,18,26,29,30,35,36,37]. Additionally, locality has an influence on the morphological features of plants [3].

Therefore, our study aimed to analyze the chemical composition of the essential oils obtained from newly developed cultivars bred for the first time under conditions of the Ukrainian central forest-steppe, to describe macromorphological features of these cultivars and to screen the antibacterial activity of the essential oils obtained from seven Lamiaceae species, namely, *Monarda bradburiana*, *Monarda didyma*, *Satureja hortensis*, *Satureja coerulea*, *Thymus vulgaris*, *Ocimum basilicum*, and *Hyssopus officinalis*.

## 2. Results and Discussion

The key morphological features of the studied cultivars are listed below.

**Figure 1:**
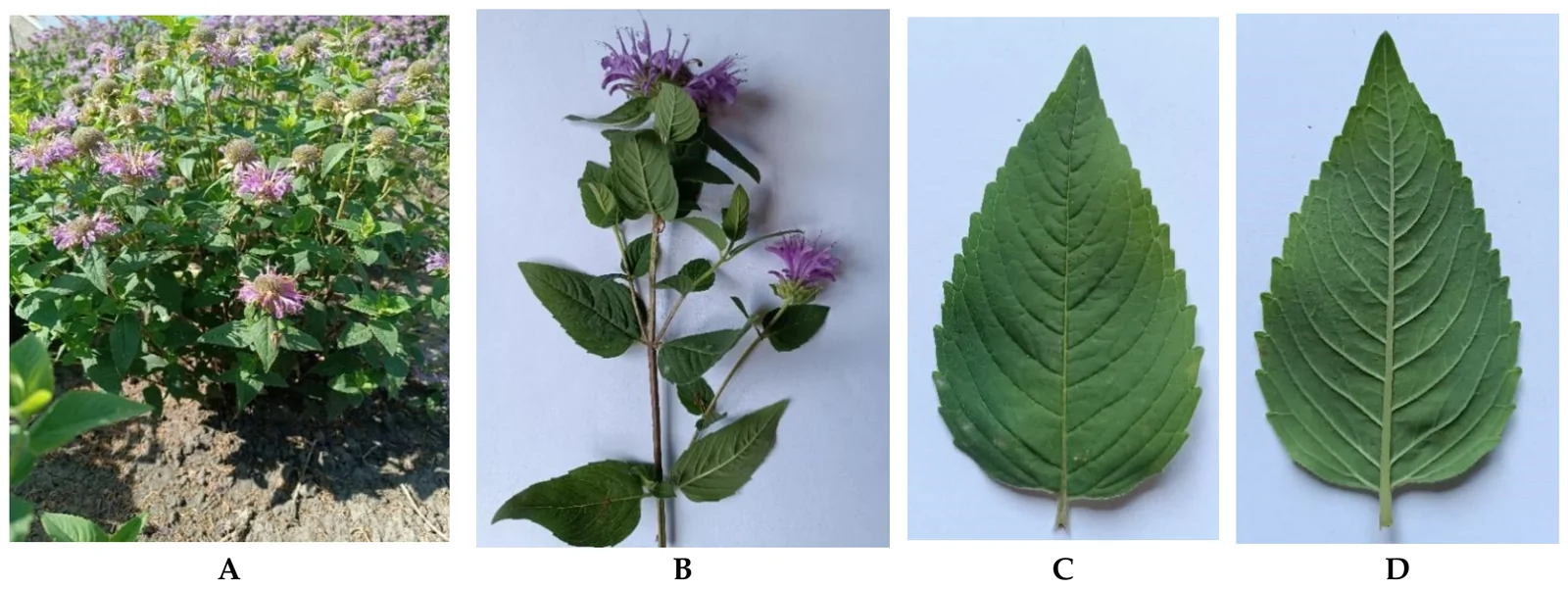
*Monarda didyma*, cultivar Md3-1. (**A**) Appearance of the plant in the phase of mass flowering; (**B**) an inflorescence; (**C**) the upper side of a leaf; (**D**) the lower side of a leaf.

*Monarda didyma* L. (Md3-1) cultivar is a perennial herbaceous plant. The cultivar was obtained from the seed generation of the *M. didyma* variety Nizhnist. In the first year of development, the height of the plants was 51–60 cm. The diameter of the plants was 37–43 cm. There were four or five first-order shoots on one plant. The leaves were 5.5–6.5 cm long, 3.0–3.5 cm wide, broadly lanceolate with serrated edges, and green with slight pubescence. The flowers were small, collected in compact spherical heads at the ends of the stems. The corolla was lilac. The diameter of the inflorescences was 4.5–6.0 cm. The fruit is a nut of brown color (Figure 1). The beginning of flowering, mass flowering and the end of flowering were observed on 25th June, 9th July, and 20th July, respectively.

**Figure 2:**
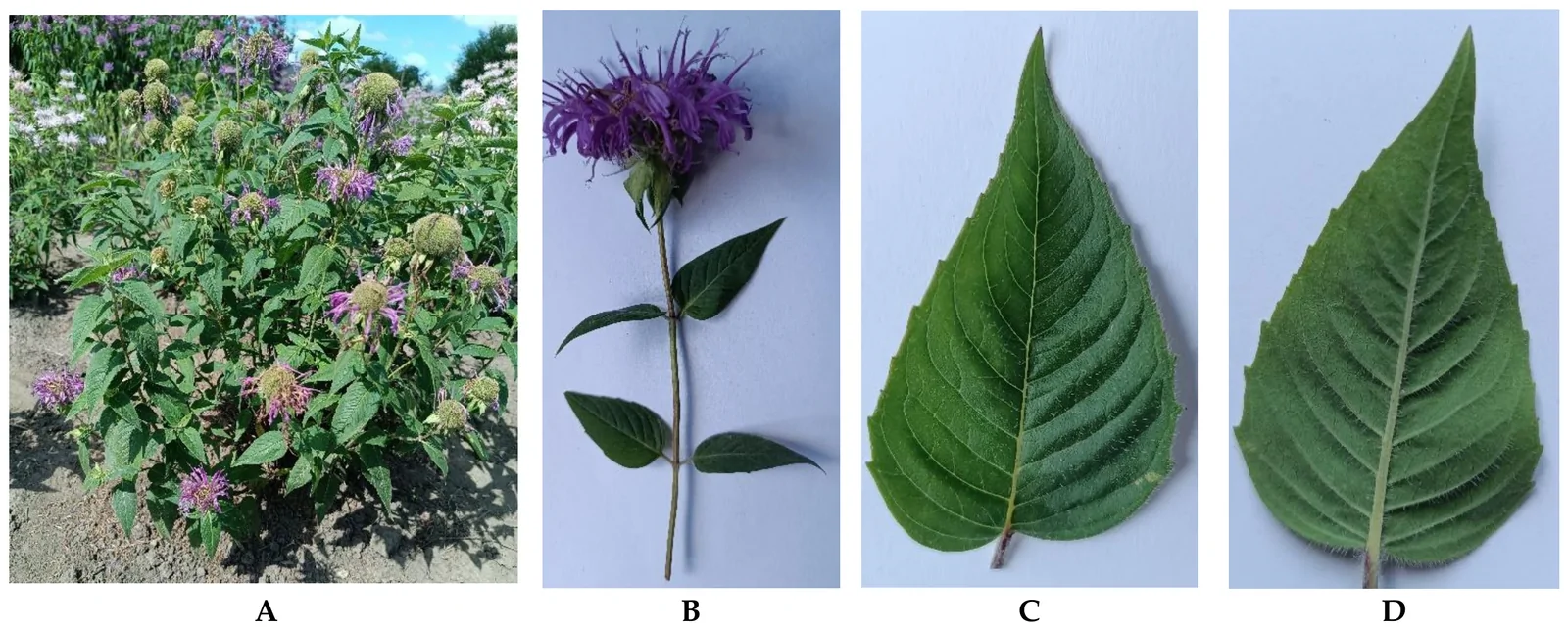
*Monarda didyma*, cultivar Md4-2. (**A**) Appearance of the plant in the phase of the end of flowering; (**B**) an inflorescence; (**C**) the upper side of a leaf; (**D**) the lower side of a leaf.

The morphological features of another cultivar of *M. didyma* (Md4-2) were slightly different despite the fact that this cultivar was obtained from the seed generation of the M. *didyma* variety Nizhnist. In the first year of its development, the height and diameter of the plants were 55–62 cm and 42–51 cm, respectively. There were between five and eight shoots of the first order on one plant. The leaves were 5.0–6.0 cm long, 3.0–3.5 cm wide, broadly lanceolate with serrated edges, dark green with slight pubescence. The flowers were small, collected in compact spherical heads at the ends of the stems. The corolla was crimson-lilac (Figure 2). The fruit is a nut of brown color. The beginning of flowering, mass flowering and the end of flowering were observed on 27th June, 15th July, and 26th July, respectively.

Compared to *M. didyma* of Italian origin, our plants were smaller. In addition, the flowers had other colors. According to Smeriglio et al. [28], *M. didyma* is an herbaceous perennial plant that grows up to 1.5 m and blooms from midsummer to autumn. Its leaves are opposite, 7.5–15 cm long, ovate–lanceolate, acuminate with serrated margins, and dark green. The inflorescence consists of a terminal head of highly compacted cymes. The flowers are two-lipped and bright scarlet red [28].

**Figure 3:**
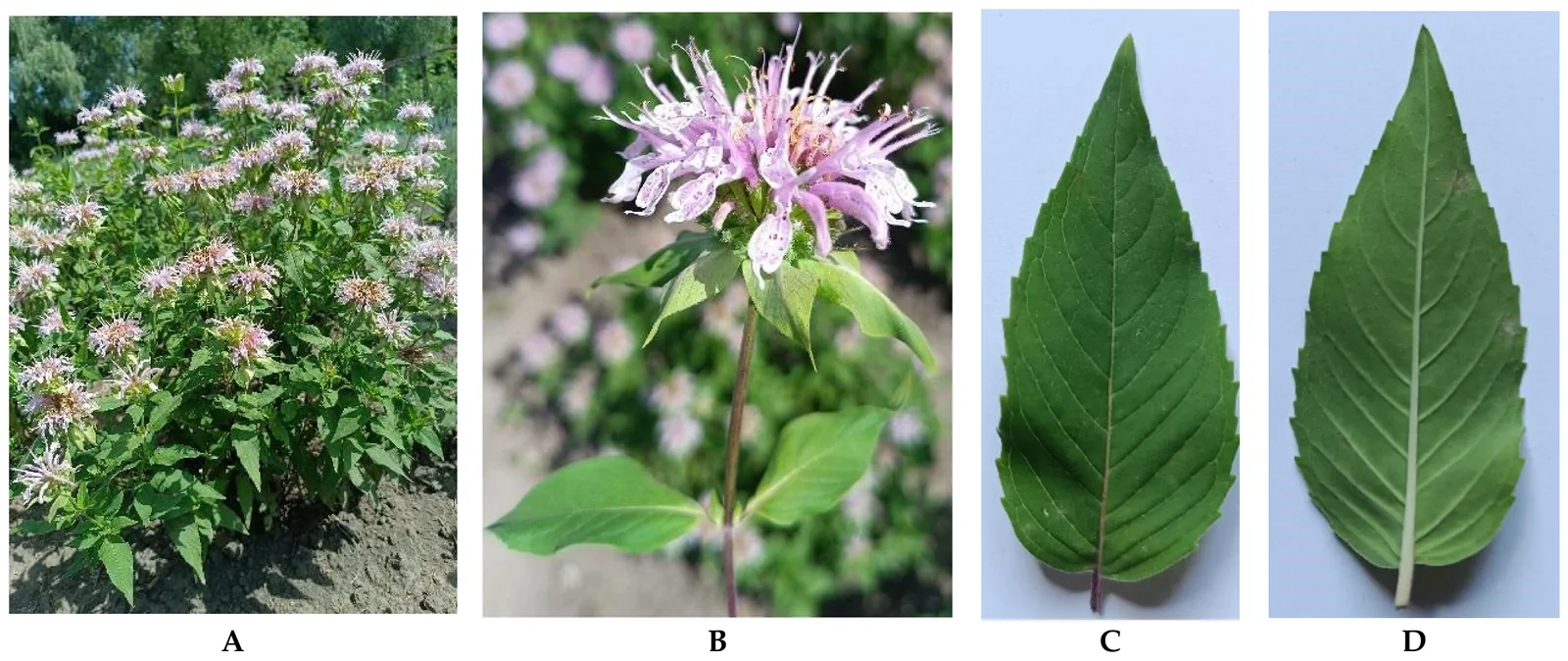
*Monarda bradbiruana*, cultivar Mb4-2. (**A**) Appearance of the plant in the phase of the end of flowering; (**B**) an inflorescence; (**C**) the upper side of a leaf; (**D**) the lower side of a leaf.

*M. bradburiana* L. C. Beck. (cultivar Mb5-09) is a perennial herbaceous plant. In the first year of its development, the height and diameter were 50–60 cm and 44–50 cm, respectively. The number of shoots of the first order was 3–4. Leaves were 8.5–9.0 cm long, 4.5–5.0 cm wide, broadly lanceolate with serrated edges, dark green with slight pubescence. The flowers were small, collected in compact spherical heads at the ends of the stems. The calyx was tubular and hairy with five teeth. The corolla was pale pink with purple spots on the blades of the lower lip (Figure 3). The fruit is a nut of brown color. The beginning of flowering, mass flowering and the end of flowering were observed on 29th June, 17th July, and 27th July, respectively.

**Figure 4:**
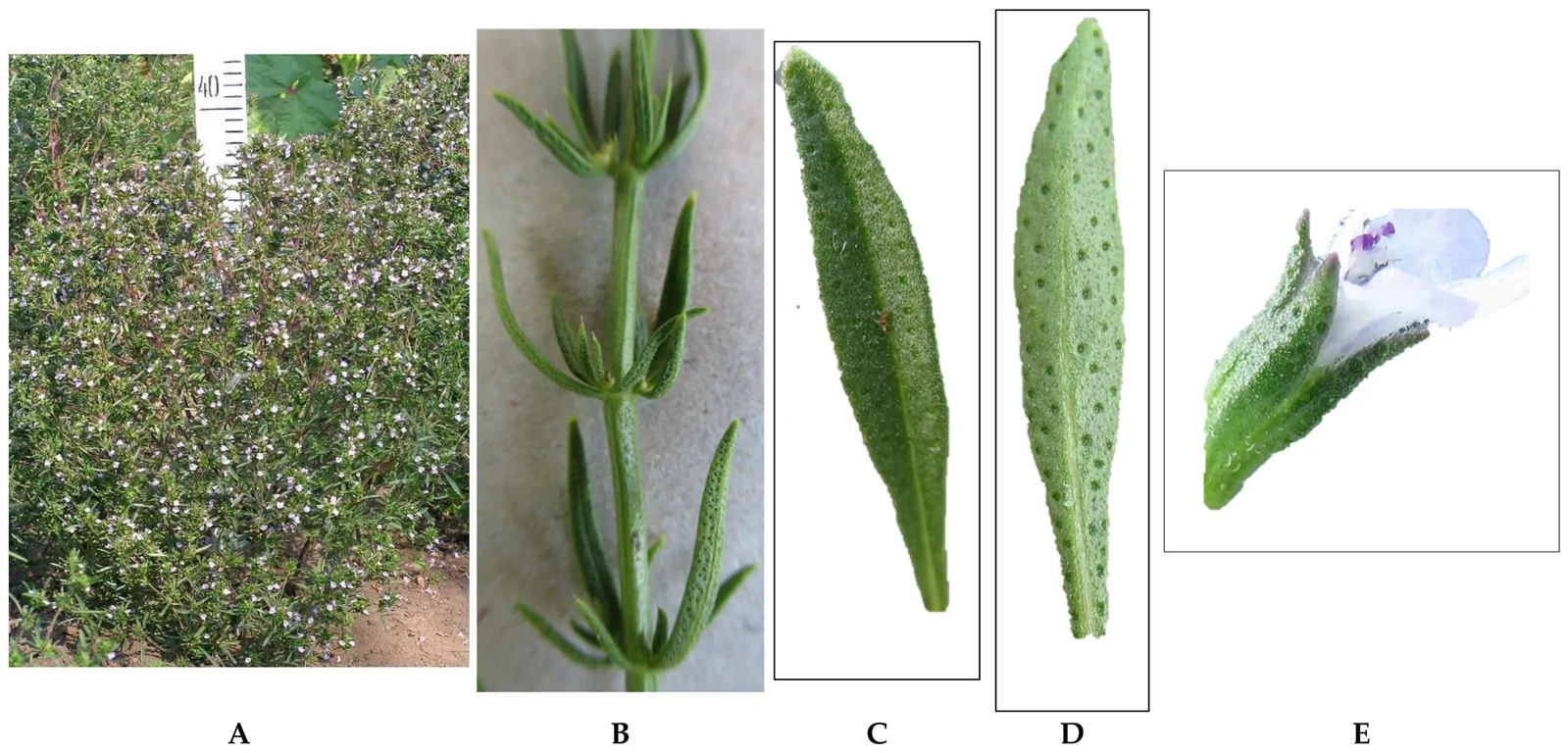
*Satureja hortensis* (Sh14-22). (**A**) Appearance of the plant in the phase of mass flowering; (**B**) a stem with branches; (**C**) the upper side of a leaf; (**D**) the lower side of a leaf; (**E**) a flower.

*S. hortensis* L. (cultivar Sh14-22). It is an annual herbaceous plant. The plant was 45–57 cm high and 45–50 cm in diameter. The stems were strongly branched, woody at the base, anthocyanin colored in the lower part and green in the upper part. The leaves were opposite, 1.5–2.5 cm long, linear-lanceolate, almost sessile, wedge-shaped and narrowed to the base. The surface of the leaves was short-haired with clearly visible essential oil glands to the naked eye. The flowers are small, light purple with purple spots, collected in axillary false rings (Figure 4). This description to a certain extent conforms to the systematic features provided by Gedik et al. [26]: annual stem, (5-)10–35 cm, leaves are linear, emucronate, 8–30 × 1–4.5 mm, verticillasters are 2–20-flowered. The corolla is lilac, purplish, or white, 4–10 mm.

Sowing was carried out in the third decade of April 2023. The beginning of budding was observed in the first decade of July. The beginning of flowering was observed on 17.07, whereas mass flowering and fruiting were observed 25.07 and 20.08, respectively. The yield of aboveground mass was, on average, 96.6 g from one plant.

**Figure 5:**
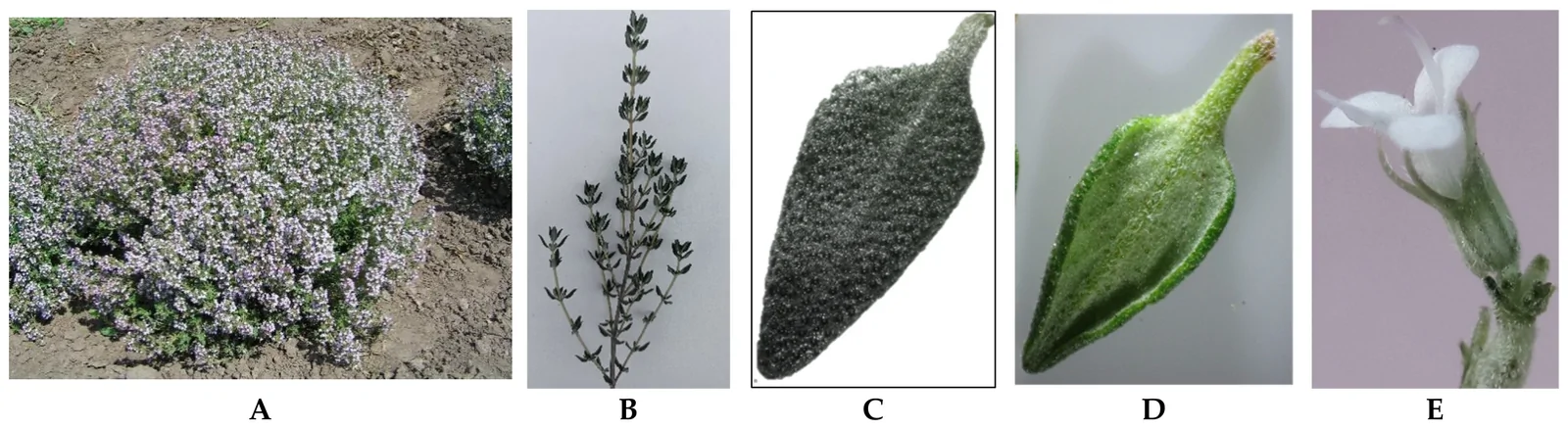
*Thymus vulgaris* (Thv16-22). (**A**) Appearance of the plant; (**B**) a stem with branches; (**C**) the upper side of a leaf; (**D**) the lower side of a leaf; (**E**) a flower.

*T. vulgaris* L. (cultivar Tv16-22) is an evergreen subshrub. In the first year of life, the plant was 43–47 cm high and 26–34 cm in diameter. Flowering stems were woody in the lower part, branched and evenly short-haired. The leaves were small, 6–7 mm long, 2.0–3.0 mm wide, short-petiolate, cobwebby pubescent on the underside, linear-elliptic, green. The edges of leaves were twisted inward. The inflorescence was spike-shaped, intermittent, consisting of between six and eight multi-flowered rings. The number of flowers in lower rings was, on average, 16–18. The flowers were small and pale pink (Figure 5).

The beginning of flowering, mass flowering, the end of flowering and fruiting were observed on 18th May, 26th May, 15th June, and 26th June. The yield of the above-ground mass of plants in the first year of development was, on average, 42 g per plant.

**Figure 6:**
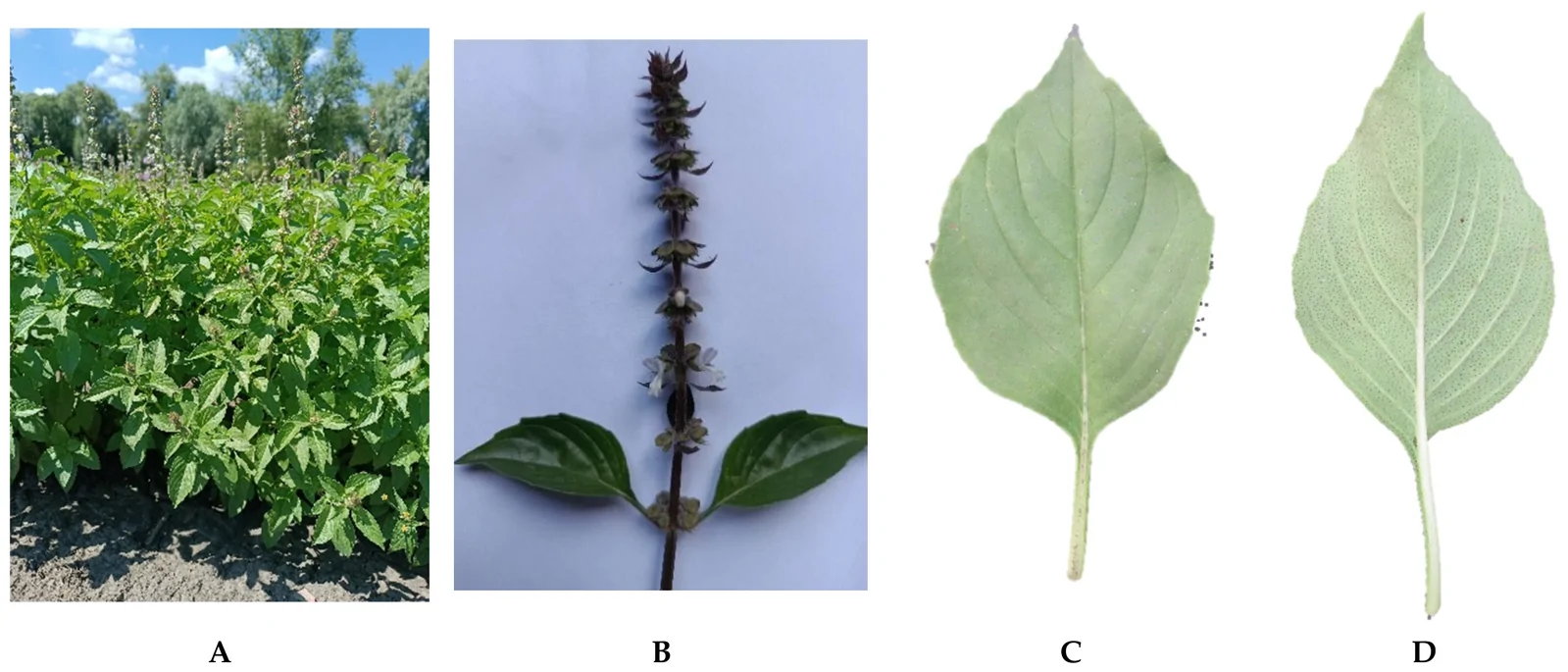
*Ocimum basilicum* (Ob17-23). (**A**) Appearance of the plant; (**B**) an inflorescence; (**C**) the upper side of a leaf; (**D**) the lower side of a leaf.

*O. basilicum* var. citriodorum L. (sample Ob17-23). The seeds were sown at the end of the second decade of May. The shoots appeared after 7 days. The plants reached a height of 70–75 cm with a diameter of 40–45 cm. The central stem was branched, tetrahedral, pubescent with short hairs, and green. The number of the first-order shoots was 8–12. The leaves were elongated-ovate, 4.5–5.5 cm long and 2.8–3.2 cm wide, and light green in color with finely toothed edges. The flowers were 1.0–1.5 cm long and white, collected in false rings in a brush in the axils of the apical leaves. The calyx was light green (Figure 6).

The beginning of flowering, mass flowering, and the end of flowering were observed on 20th July, 7th August, and 1st September, respectively. The yield of above-ground mass was, on average, 85.5 g from one plant.

**Figure 7:**
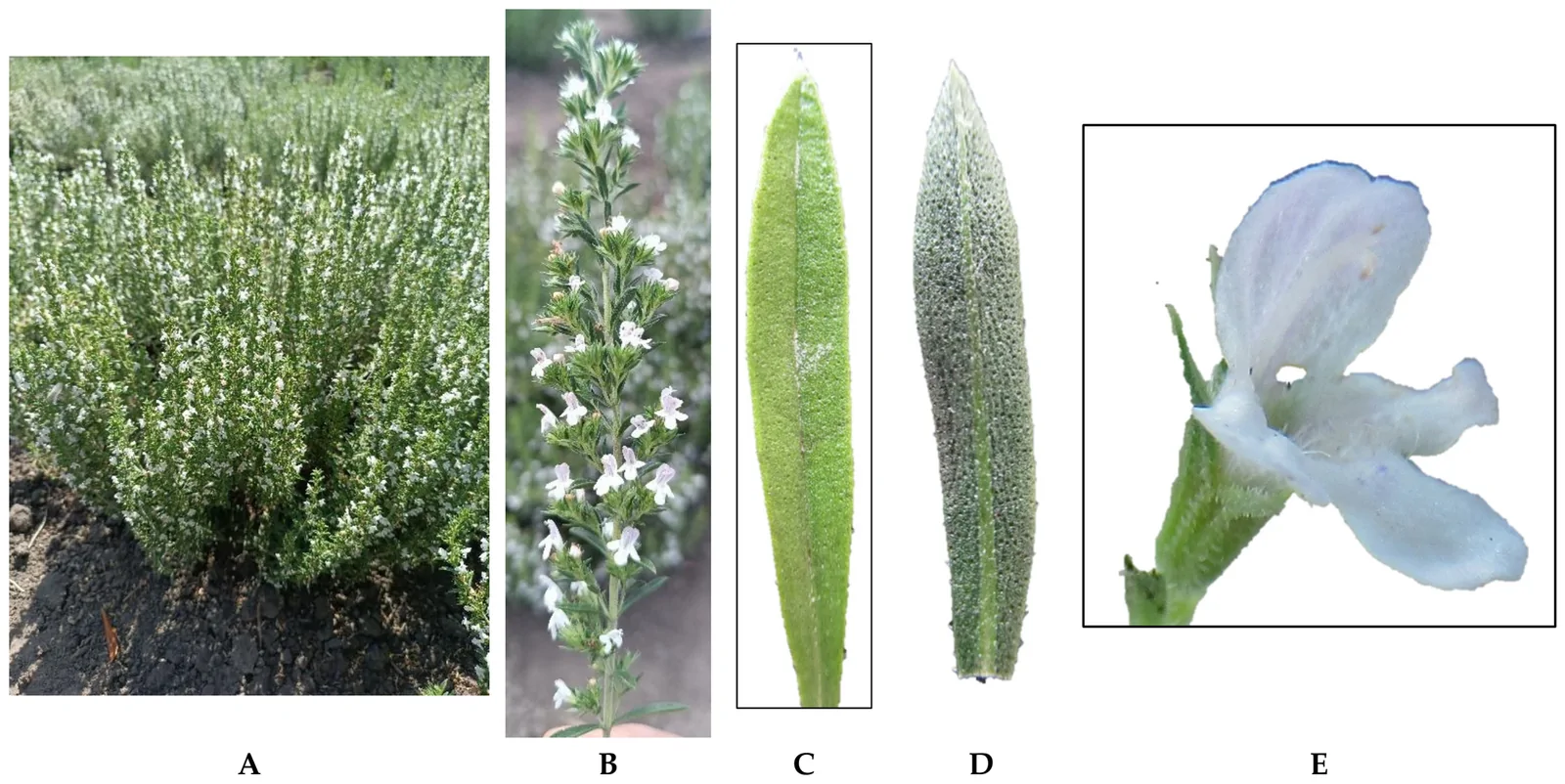
*Satureja coerulea* (Sc19-23). (**A**) Appearance of the plant; (**B**) an inflorescence; (**C**) the upper side of a leaf; (**D**) the lower side of a leaf; (**E**) a flower.

*S. coerulea* Janka, cultivar Sh19-23. It is a compact perennial subshrub. In the first year of life, the plant was 40–45 cm high and 43–47 cm in diameter. The stems were erect, almost rounded, light brown, densely leafy, and branched in the upper part. The cauline outer leaves were 1.5–2.0 cm long and 0.4–0.5 cm wide, whereas the cauline inner leaves were smaller, 1.3–1.6 cm long and 0.3–0.4 cm wide. The flowers were 0.9–1.0 cm, white with a bluish tint, and collected in raceme-shaped paniculate inflorescences 12–14 cm long. The calyx was short-tubular and light green. Its teeth were styloid-linear and took up almost half of the length of the tube. The fruit was a light brown nut (Figure 7).

This description is partly in line with the characteristic features provided by Satil et al. [33], because there are main differences in the size of cauline leaves and the color of the corolla. *S. coerulea* is a perennial suffruticose plant of 6–25 cm. The cauline leaves are oblong, linear and pale green. The outer leaves are large, 16–11 × 2.0–2.3 mm, whereas inner leaves are smaller, 6–4 × 1.0–0.4 mm, and sessile. Inflorescences are generally long, 3–12 cm, mostly congested at the apex. The calyx is light green, 2.5–4–5 mm. It has five teeth. The teeth are 1/3–2/5 x calyx, lanceolate subulate, and glabrous. The corolla is light purple, 6–9 mm. The nutlets are light brown, 0.6–1.2 × 0.3–0.8 mm, and obovate. This plant flowers in August–October [33].

The beginning of mass flowering was observed at the end of the second decade-third decade of August, whereas fruiting was in September. The yield of above-ground mass was, on average, 80 g from one plant.

**Figure 8:**
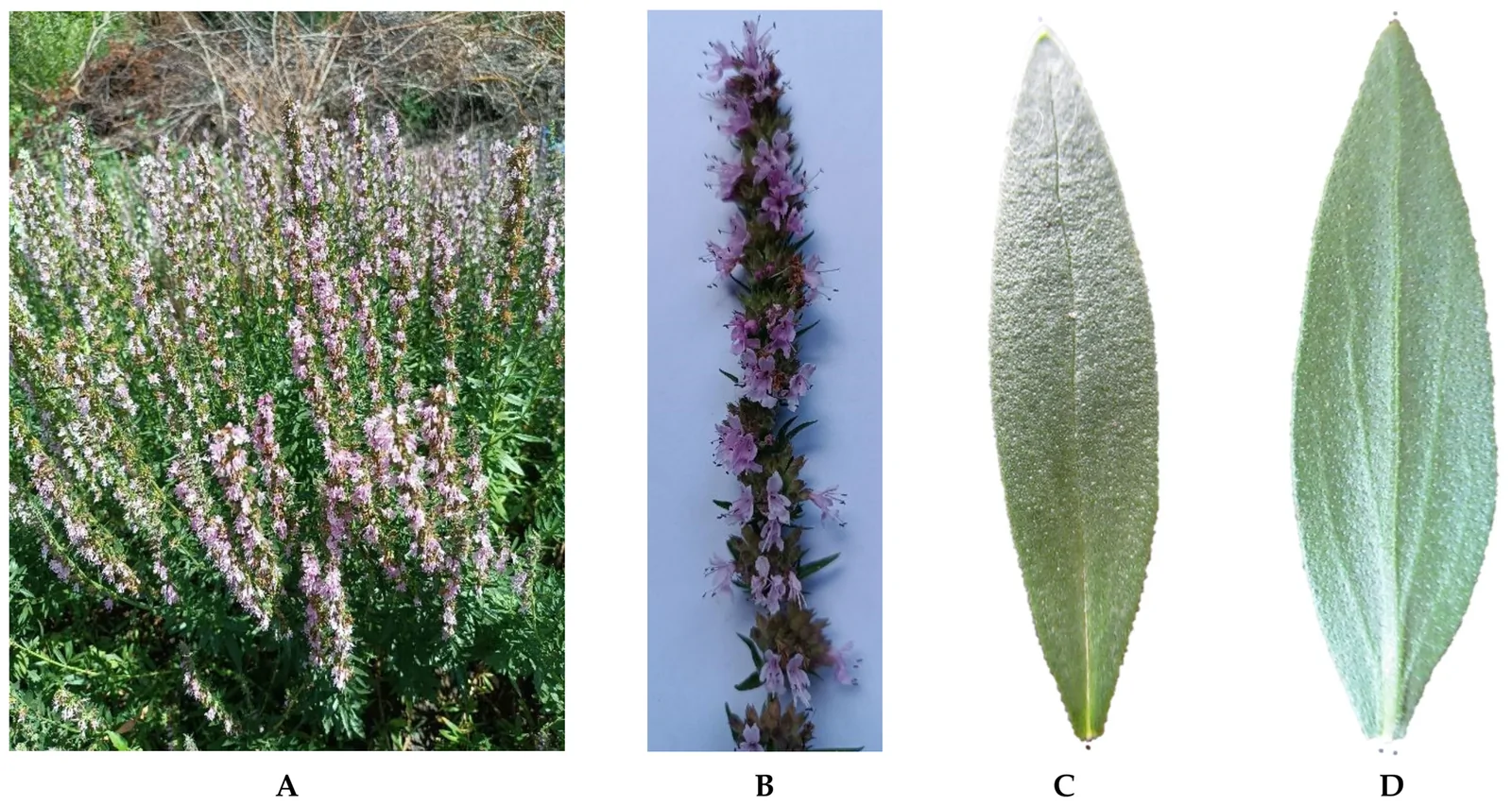
*Hyssopus officinalis* (Ho20-7-18). (**A**) Appearance of the plant; (**B**) an inflorescence; (**C**) the upper side of a leaf; (**D**) the lower side of a leaf.

*H. officinalis* L., cultivar Ho20-7-18, is a perennial semi-shrub. The plants of the first year of growth had a semi-spreading form and reached a height of 55–60 cm with a diameter of 25–30 cm. The stem was tetrahedral and woody at the base. The number of first-order shoots was 12–14. The length of the first-order shoot was 15–25 cm. The leaves were sessile, linear-lanceolate, opposite, entire. The leaf blade was densely pubescent on both sides, 3.0–3.5 cm long and 0.7–0.9 cm wide. In the first year of development, the plant bloomed. The flowers were small, pink, collected in the axils of the leaves in false semi-rings and formed a sawdust-like inflorescence in the upper part of the stem (Figure 8). The fruit was a nutlet of oblong-ovate shape, trihedral and black. In plants of the first year of development, the beginning of flowering was observed on 10th August, whereas mass flowering and the end of flowering occurred on 20th August and 15th September.

Our description is partly in line with the morphological properties of *H. officinalis* growing in Kazakhstan. The leaves were lanceolate, short-petiolate, and short-hairy. The inflorescences were long and spicate. The flowers sit in the axils of the upper leaves. The purple calyx was 5–6 mm long, with five teeth, increased to one-third of the total length of the calyx, with short hairs along the veins. The purple corolla was 10–15 mm long, two-lipped, with an upper lip with a notch, shorter than the lower lip. The lower lip had a well-defined, downwardly bent middle lobe [35]. The general description of *H. officinalis* is also provided by Nurzynska-Wierdak [36]. The height of the plant is 30–70 cm. The leaves are very fragrant, small, narrow, lancet-shaped, and pointed. There exist different colous of zygomorphic flowers (purple-dark blue, pink, white and sometimes red) gathered in a magnificent, peak inflorescence [36].

### 2.1. Yield of the Essential Oils

In general, the yield in the distillation process is usually low and it depends on the species, variety, phase of development [16,30,36,38] and locality [29,36]. It is worth noting that the technique of obtaining and a plant part have a great impact on the yield (traditional distillation, extraction, microwave-assisted hydrodistillation, ohmic-assisted distillation, etc.) as well [16,19,38].

As far as the yields of the essential oils are concerned, they were in the range of 0.07–0.70% of fresh weight or 0.32–2.40% of dried weight (Table 1).

The percentage yields of eight essential oils from the aboveground parts of the tested plants allow reaching the conclusion that *O. basilicum* cultivated in Ukrainian forest-steppe is a very poor essential oil representative of the Lamiaceae family, similar to *Salvia verbenaca* [4].

Özcan and Chalchat [29] reported a yield 1.25% for the essential oil of *O. basilicum* of Turkish origin from air-dried overground parts, while Joshi [18] found the yield of 0.21% (*v*/*w*) for the essential oil of *O. basilicum* of Indian origin, which was obtained from the fresh material. However, according to Katekar et al. [38], the yields for *O. basilicum* were in the range of 1.25% to 2.1% with respect to the dried material. In addition, Özcan and Chalchat stated that the yield for *O. basilicum* can range from 0.15% to 1.59% depending on the locality and season of gathering this plant [29].

Fani and Kohanteb asserted a yield of 1.6 ± 0.34 g of the essential oil per 100 g of dried leaves for *T. vulgaris* of Iranian origin [11]. According to Katekar et al. [38], the yield for *S. hortensis* was 3.1%. Azaz et al. found the yield 0.6% from the air-dried aerial parts of *S. coerulea* of Turkish origin [27]. According to Nurzyńska-Wierdak [36], *Hyssopi* herba contains 0.3–1.8% of essential oil.

Lawson et. al. [32] reported a 3.52% (*w*/*w*) yield of the essential oil obtained by hydrodistillation from the dried leaves of *M. bradburiana* grown in Minnesota (USA). The leaves and inflorescences of *M. bradburiana* grown in Poland (Warsaw) gave a yield of 0.82 mL/100 g and 0.05 mL/100 g of the dried material [19]. Therefore, yields depend on a part of the plant.

A yield (*v*/*w*) of 2.55 and 2.61 mL/kg dry weight was measured for the essential oil of *M. didyma* of Italian origin that was obtained with steam distilled in a Clevenger-type apparatus for 3 h [12,21]. *M. didyma* from Quebec gave a yield 0.49% [9]. However, the yields of *M. didyma* of Polish origin ranged from 2.46% to 3.71% of dry weight depending on a part of the plant (leaves, flowers or herb) [1].

According to the classification proposed by Radosavljević et al. [39], our cultivars belong to different groups. This classification divides plants into four classes for the yield of essential oil: poor yield, <1.3%; regular yield, 1.3–1.8%; good yield, 1.81–2.3%; and excellent yield, more than 2.3%. Our cultivars of *O. basilicum*, *H. officinalis*, and *S. coerulea* belong to cultivars with poor yields of essential oil, whereas *M. bradburiana* and *T. vulgaris* belong to cultivars with regular yields; *M. didyma* can be a cultivar with poor or good yields depending on a cultivar. *S. hortensis* refers to cultivars with excellent yields.

We can observe that under conditions of the Ukrainian forest-steppe, some cultivars give lower yields of essential oils compared to cultivars grown on more southern territories. For instance, *O. basilicum* (0.32%) vs. the same species of Turkish origin (1.25%) [29]. However, *O. basilicum* of Indian origin gave a higher yield (0.21% fresh mass) [18].

Our cultivar of *M. bradburiana* showed (1.65%) vs. *M. bradburiana* grown in Minnesota (3.52%) [32]. The leaves of *M. bradburiana* grown in Warsaw gave a lower yield (0.82 mL/100 g). Warsaw is a more northern city compared to the Cherkasy region. The yields of essential oil were significantly higher for *M. didyma* of Italian origin (2.55% [12] and 2.61% [21] vs. our 0.99% and 2.10%), whereas the yield of essential oil for *M. didyma* from Quebec was lower (0.49%) [9].

The generalized information about flowering, average yield of mass per one plant, yields of essential oil, and date of distillation are provided in Table 1.

### 2.2. Chemical Composition and Antibacterial Activity of the Essential Oils

The results of GC-MS are provided in Table 2.

In general, *Monarda* essential oil is a light-yellow liquid with a pleasant floral-spicy aroma, featuring thyme and lemon notes. It is found in stems, inflorescences, and leaves [1,30]. It was revealed that in sample Md3-1 of *M. didyma*, thymol was a principal component (58.5%), followed by 4(10)-thujene (sabinene) (7.0%), *p*-cymene (5.8%), 7-octen-4-ol (4.7%), carvacrol (4.2%) and γ-terpinene (4.1%). Our results are in line with the studies carried out by Cichosz et al. [1], Ricci et al. [21], and Dudchenko et al. [30].

Recently, Cichosz et al. [1] have analyzed, using GC–MS, three samples of the essential oil of *M. didyma* originating from Lublin, Poland. Three samples were obtained from the leaves, flowers, and herb, respectively. It was revealed that thymol was a key component (51.6–68.6%); namely, this chemotype was the thymol one. *o*-Cymene and γ-terpinene were the second and third components, but they were in significantly lesser amounts, at 3.7–5.8% and 2.9–4.5%, respectively. The MIC values ranged from 0.25 to 0.5 mg/mL for such Gram-positive bacteria as *Bacillus subtilis* ATCC 6633, *B. cereus* ATCC 10876, *B. cereus* ATCC 13061, and *Micrococcus luteus* ATCC 10240, whereas the MIC values ranged from 0.5 to 2.0 mg/mL for such Gram-positive bacteria as *Staphylococcus* spp. and *Enterococcus* spp. The herb essential oil showed the highest susceptibility against *S. aureus* ATCC 43300, with an MIC of 0.5 mg/mL. The Gram-negative bacteria showed a sensitivity to the tested essential oils at the level of 0.25–0.5 mg/mL (the strains of *Salmonella enteritidis* ATCC 13076; *S. typhimurium* ATCC 14028; *Proteus mirabilis* ATCC 12453; *Bordetella bronchiseptica* ATCC 4617; different strains of *Escherichia coli* and *Klebsiella pneumoniae*) [1].

The essential oil of *M. didyma* L., variety Nizhnist (the Ukrainian origin), contained 70.4% of thymol, 7.2% of γ-terpinene, 4.6% of methyl carvacrol, 4.5% octenol-3, 3.7% of *p*-cymene, and 2.4% of carvacrol [30]. It seems that cultivar Md3-1 keeps the initial features of the Nizhnist variety. The essential oil of the flowering aerial parts of *M. didyma* cultivated in central Italy and analyzed by GS-MS contained thymol (59.3%), *p*-cymene (10.3%), terpinolene (δ-terpinene) (9.2%), δ-3-carene (4.4%), myrcene (3.7%), and camphene (3.4%) [21]. It is worth mentioning that high contents *p*-cymene and γ-terpinene have negative effects on the odor and biological properties of *Monarda* essential oils and, consequently, make these essential oils inappropriate for the perfume and cosmetic industries [30].

In contrast to the sample Md3-1, it was revealed that in the sample Md4-2 carvacrol was the principal component (69.9%), followed by 7-octen-4-ol (4.8%), *p*-cymene (4.3%), and γ-terpinene (3.6%); namely, this sample of *M. didyma* belonged to the carvacrol chemotype. Our studies are in agreement with the studies performed by Côté et al. [9].

The *M. didyma* essential oil of Canadian origin (the province of Quebec) was rich in carvacrol. It contained carvacrol (49.0%), γ-terpinene (16.9%), *p*-cymene (7.6%), thymol (6.2%), carvacrol methyl ether (4.2%), 1-octen-3-ol (3.1%), α-terpinene (2.8%), myrcene (2.3%), α-thujene (1.4%), and limonene (1.0%). The essential oil demonstrated MIC values of 47 ± 8 µg/mL and 35 ± 4 µg/mL for two Gram-positive bacteria, *Staphylococcus aureus* ATCC 25923 and *Clostridium perfringens*, respectively, and 87 ± 8 µg/mL for the Gram-negative bacterium *Escherichia coli* ATCC 25922 [9].

In another study, the essential oil from the *M. didyma* plants growing in L’Acadie (45°17′ N; 73°20′ W) (Canada) contained carvacrol (15.2%), γ-terpinene (15.6%), thymol (41.2%), α-terpinene (4.6%), myrcene (2.5%), and α-thujene (2.6%) [10]. This chemotype was classified as the thymol chemotype.

In the essential oil of *M. didyma* grown in central Italy, there were 2.6% of linalool, 2.7% of α-terpinene, 5.5% of 1-octen-3-ol, 5.2% of thymol methyl ether, 6.4% of carvacrol methyl ether, 8.8% of γ-terpinene, 12.3% of thymol, 19.3% of *p*-cymene, and 19.5% of carvacrol [12]. It looks like that this chemotype is a mixed thymol, *p*-cymene, and carvacrol chemotype. However, the authors referred this chemotype to the carvacrol one.

The principal components in the essential oil of *M. bradburiana* (sample Mb5-09) were carvacrol (63.6%), 1-octen-3-ol (7.7%), and γ-terpinene (7.1%), *p*-cymene (6.3%), and thymol (0.7%). It is considered that this plant belongs to the carvacrol chemotype.

Our studies are not in line with studies of Lawson et al. [32] concerning the most principal components thymol/carvacrol and yield of the essential oil. The main components in the leaf essential oil of *M. bradburiana* cultivated in south Alabama (USA) were thymol (60.1%), *p*-cymene (9.8%), carvacrol (5.7%), (–)-1-octen-3-ol (4.9%), and γ-terpinene (4.7%), α-terpinene (3.3%), and (E)-*β*-caryophyllene (1.5%) [32].

Whereas the leaf essential oil of *M. bradburiana* of Polish origin contained thymol (49.9%), *p*-cymene (18.7%), carvacrol (5.5%), and γ-terpinene (2.5%) [19]. It looks like that these samples of American [32] and Polish origin [19] are similar for the chemical composition of their essential oils. We suppose that these samples of *M. bradburiana* belong to the thymol chemotype. In addition, the essential oil of *M. bradburiana* of Polish origin showed the following MIC 0.313, 0.313, 0.625, 0.625, 0.625 and 0.313 μL/mL for *S. aureus*, *L. monocytogenes*, *E. coli*, *C. jejuni*, *A. fumigatus,* and *C. albicans*, respectively [19].

The screening microbiological studies showed that sample Md3-1 was more active against *S. aureus* (26.3 ± 0.2 mm) compared to sample Md4-2 (20.0 ± 0.3 mm) and Mb5 (23.0 ± 0.2 mm), whereas that sample Md3-1 was more active against *E. coli* (32.0 ± 0.3 mm) compared to sample Md5-09 (26.0 ± 0.2 mm). Moreover, the cultivar Mb5-09 possessed strong activity against the clinical strain of *Klebsiella pneumoniae* (Table 3).

The diameter of the inhibition zone of the essential oil of *M. didyma* against twenty carbapenem-resistant *Klebsiella pneumoniae* strains was in the range of 29.67 ± 0.47 mm to 24.67 ± 0.47 mm. The composition was as follows: thymol (63.8%), 2-lignocaine, β-cinnamene, pinene, terpene, cyclamen, terpinene, and δ-3-carene [6].

It was revealed that *Monarda didyma* (cultivar ‘Cambridge Scarlet’) possessed strong antimicrobial activity for 10 µL of the essential oil introduced into the wells against *S. aureus* ATCC25923 (diameter of inhibition zone was 30.0 ± 0.50 mm), *S. aureus* clinical strain (25.0 ± 0.25 mm), *C. albicans* ATCC885–653 (21.0 ± 0.50 mm), and *C. albicans* clinical strain (22.0 ± 0.55 mm); moderate activity against *S. pyogenes* ATCC 19615 (18.0 ± 0.25 mm), *S. pyogenes* clinical strain (17.0 ± 0.80 mm), *E. coli* ATCC 25922 (15.0 ± 1.00 mm), and *E. coli* clinical strain (14.0 ± 1.00 mm), and weak activity against *Enterococcus faecalis* ATCC 29212 (10.0 ± 0.30 mm) and *E. faecalis* clinical strain (9.0 ± 0.25 mm). The clinical strains of the bacteria and yeasts were isolated from the oral cavities of patients suffering from inflammatory periodontium and pharynx. This essential oil had the following composition: thymol 47.5%, eucalyptol 17.8%, α-terpineol 8.3%, isothymol methylether 5.9%; namely, this cultivar belonged to the thymol chemotype [40].

It is worth mentioning that the results of microbiological studies significantly differ across publications. For instance, the MIC values of the essential oils of *M. didyma* of Polish origin for five different strains of *S. aureus* were in the range of 0.5 to 1 mg/mL [1], whereas in another study for the essential oil from the freshly harvested aerial parts of *M. didyma* of Canadian origin was 47 ± 8 µg/mL [9].

It was revealed that our sample of the *S. hortensis* essential oil belonged to the carvacrol chemotype, because carvacrol was a principal component (66.3%), followed by γ-terpinene (16.0%) and *p*-cymene (6.2%). In general, there are two described chemotypes of *S. hortensis*: thymol and carvacrol ones [8,26].

Mahboubi and Kazempour [8] identified thymol (28.2%), *p*-cymene (19.6%), γ-terpinene (16%), carvacrol (11%), β-pinene (4.5%), sabinene (4.4%), α-pinene (2.7%), and 4-terpineol (1.6%) in the aerial parts of *Satureja hortensis* grown in Kashan (Iran). The sum of thymol and carvacrol was 39%. The MIC of the essential oils of *S. hortensis* was in the range of 0.125 to 4 μL/mL for such microorganisms as *S. aureus* ATCC 25923, *S. saprophyticus* ATCC 15305, *S. epidermidis* ATCC 14490, *Enterococcus faecalis* ATCC 29212, *E. faecium* ATCC 25778, *Streptococcus sanguis* ATCC 10556, *S. salivarius* ATCC 9222 (0.125 μL/mL), *K. pneumoniae* ATCC 10031 (0.5 μL/mL), *E. coli* ATCC 8739, *Salmonella typhimurium* ATCC 14028, *Proteus vulgaris* RI 231 (0.125 μL/mL), *Enterobacter aerogenes* NCTC 10009, *Shigella dysantri* RI 366 (0.125 μL/mL), *S. flexeneri* NCTC 8516 (0.125 μL/mL), *Serratia marcescens* ATCC 13880 (0.25 μL/mL), whereas the MIC was in the range of 0.06 to 1 μL/mL for the fungi *C. albicans* ATCC 10231, *C. glabrata* ATCC 90030, *A. flavus*, *A. niger* ATCC 16404, and *A. parasiticus* ATCC 15517. The least sensitive microorganism to the essential oil was *P. aeruginosa* (MIC 8 μL/mL) [8].

Gedik et al. [26] examined three genotypes of *S. hortensis* of Turkish origin, which grew in three different locations. Eighteen components were identified in the Nurhak genotype. Thymol was the main component (41.0%), which was followed by *γ*-terpinene (17.2%), cymene (16.6%), β-pinene (5.5%), carvacrol (5.3%), and α-pinene (4.8%), whereas fourteen components were identified in the Baskonus genotype. The Baskonus genotype stood out in a high abundance of carvacrol (64.1%), which was followed by *γ*-terpinene (18.0%), cymene (7.8%), and α-pinene (1.7%). Sixteen components were identified in the Kapıdere genotype. Carvacrol was also the main component (40.0%), followed by *γ*-terpinene (32.4%), cymene (13.0%), α-pinene (1.9%), and α-terpinene (2.0%). We suppose that the Nurhak genotype belonged to the mixed chemotype with domination of thymol (the thymol/carvacrol chemotype), whereas the other two genotypes belonged to the carvacrol chemotype.

Our analyses showed that carvacrol (49.4%) was the key component in the essential oil of *S. coerulea* (Sh14). Other principal components were identified as *γ*-terpinene (13.6%), thymol (12.4%), *p*-cymene (3.9%), and 2-carene (2.3%). We suppose that this chemotype is carvacrol-thymol.

Shanaida et al. [2] identified such components in the *S. coerulea* herb of Ukrainian origin as thymol (33.2%), *o*-cymene (14.4%), terpinen-4-ol (8.3%), *γ*-terpinene (7.3%), carvacrol (5.1%), 1-octen-3-ol (4.5%), linalool (2.6%), (+)-2-carene (2.1%) and borneol (1.6%). However, the essential oil of the dried aerial parts of *S. coerulea* of Turkish origin was rich in sesquiterpenes. It contained *β*-caryophyllene (10.6%), caryophyllene oxide (8.0%), borneol (6.3%), germacrene D (4.7%), limonene (4.3%), 4-bourbonene (3.6%), and spathulenol (3.0%). This essential oil demonstrated MIC at the level of 125 µg/mL for *E. coli* ATCC 25292, *S. aureus* ATCC 6538, *P. aeruginosa* ATCC 27853, *E. aerogenes* NRRL 3567, *Proteus vulgaris* NRRL 123, and *Salmonella typhimurium* NRRL 4420 [27].

In general, Gedik et al. [26] distinguish two chemotypes in the *Satureja* and *Thymus* genera: carvacrol and thymol chemotypes. It is worth noting that there is a complicated situation with the identification of cymene isomers by GC-MS in publications. For instance, Gedik et al. [26] did not indicate the type of cymene isomers in their study related to *S. hortensis* and *S. cuneifolia*. Shanaida et al. [2] identified *o*-cymene in the essential oil of *S. coerulea* of Ukrainian origin. The same situation with *o*-cymene in the essential oil from *M. didyma* of Polish origin [1].

Here, some studies connected to the biosynthesis of thymol, carvacrol and *p*-cymene are worth attention. Krause et al. [20] showed that *p*-cymene is a frequent component of plant essential oils, including Lamiaceae species, such as thyme and oregano. Moreover, thymol and carvacrol are formed in a cascade of the following reactions. The first reaction leads to the cyclisation of geranyl diphosphate to γ-terpinene. Then γ-terpinene is oxidised by cytochrome P450 monooxygenases of the CYP71D subfamily to produce unstable cyclohexadienol intermediates, which are dehydrogenated by a short-chain dehydrogenase/reductase to the corresponding ketones. Later, these ketones are converted into thymol or carvacrol through tautomerization. Furthermore, all the CYP71D enzymes produce *p*-cymene in the in vitro assay in the absence of the short-chain dehydrogenase/reductase. Therefore, the ratio of *p*-cymene to thymol or carvacrol in the plant may depend on the relative activities of the two enzymes (CYP71D and short-chain dehydrogenase/reductase) and physical distance between them in the cell. If the dehydrogenase is closely associated with cytochrome P450S, the likelihood of the *p*-cymene biosynthesis will be lower. If this dehydrogenase is absent, *γ*-terpinene is oxidized to *p*-cymene. Additionally, biosynthesis of thymol or carvacrol depends on the type of enzymes of the CYP71D subfamily [20]. However, Poulose and Croteau [41] assert that thymol is biosynthesized by aromatizing *γ*-terpinene to *p*-cymene, followed by hydroxylation of *p*-cymene. In any case, *γ*-terpinene, *p*-cymene, thymol, and carvacrol are interrelated compounds in some essential oils. They are supposed to be in various ratios in essential oils depending on the activity of various enzymes.

The main components of the essential oil of *T. vulgaris* were carvacrol (4.5%), linalool (4.7%), *p*-cymene (7.9%), γ-terpinene (11.2%), linalool (4.7%), and thymol (49.9%). We suppose that this sample of *T. vulgaris* belongs to the thymol chemotype. The screening microbiological studies demonstrated pronounced antibacterial activity of the investigated *T. vulgaris* essential oil. Among the tested Gram-positive microorganisms, *S. aureus* ATCC 25923 was the most susceptible, with an inhibition zone of 25.0 ± 0.2 mm, whereas the inhibition zone for *S. epidermidis* ATCC 12228 was 20.0 ± 0.3 mm. Considerable antimicrobial activity was also observed against the Gram-negative bacterium *E. coli* ATCC 25922, with an inhibition zone of 22.0 ± 0.3 mm. In contrast, no growth inhibition was observed for *P. aeruginosa* ATCC 27853 or *K. pneumoniae* No. 43 under the experimental conditions.

Our findings concerning the high susceptibility of *S. aureus* and *E. coli* are consistent with some previously published data. Borugă et al. [17] investigated a thymol-rich *T. vulgaris* essential oil containing thymol (47.6%), *γ*-terpinene (30.9%), and *p*-cymene (8.4%), which belonged to the thymol chemotype. This composition was comparable to that of our sample, particularly with respect to thymol (49.9%) and *p*-cymene (7.9%). Using the disk-diffusion method, the authors reported inhibition zones from 23.93 ± 0.33 to 31.40 ± 0.47 mm against *S. aureus* ATCC 25923 and from 14.63 ± 0.36 to 34.99 ± 0.19 mm against *E. coli* ATCC 25922, depending on the amount of the essential oil applied (5–20 μL). These findings are in agreement with the pronounced activity observed in our study against the same strains, with inhibition zones of 25.0 ± 0.2 and 22.0 ± 0.3 mm, respectively.

Similar results were reported by Galovičová et al. [7] for a Slovak-origin *T. vulgaris* essential oil of the thymol chemotype, which exhibited a chemical profile particularly close to that of our sample. The essential oil was obtained from Hanus, s.r.o. (Nitra, Slovakia) and produced by steam distillation of the partially dried stalks. It contained thymol (48.1%), *p*-cymene (11.7%), 1,8-cineole (6.7%), γ-terpinene (6.1%), carvacrol (5.5%), and linalool 4.4%. The authors reported moderate to very strong antimicrobial activity, with inhibition zones ranging from 9.89 to 22.44 mm from the saturation of the disks with 10 µL of the essential oil. *S. aureus* was among the most susceptible microorganisms and exhibited one of the lowest MIC values, with an inhibition zone of 10.7 ± 1.0 mm [7]. These findings support the pronounced anti-staphylococcal activity observed in our study and suggest that the high thymol content, together with carvacrol and other monoterpenes, may substantially contribute to the antibacterial properties of the investigated essential oil. It is worth mentioning that Galovičová et al. [7] used a somewhat different classification of antimicrobial activity. The antimicrobial activity was very strong if the inhibition zone diameter was above 15 mm, moderate activity if the inhibition zone diameter was in the range from 10 to 15 mm, and weak activity if the inhibition zone diameter ranged from 5 to 10 mm [7].

The results of the agar disk diffusion assay demonstrated that various concentrations (256 to 2 mg/mL) of the *T. vulgaris* oil against the tested isolates had the inhibition zones ranging from 7.5 ± 0 to 42 ± 0.8 mm in diameter. At concentrations from 64 to 256 mg/mL, *S. pyogenes* was the most sensitive as the inhibition zones ranged from 21.1 ± 0.8 to 42 ± 0.8 mm, whereas *Streptococcus mutans* (the main etiologic agent of dental caries), and *Porphyromonas gingivalis* showed the inhibition zones from 18.9 ± 1 mm to 38.1 ± 1 mm and from 9.5 ± 0.5 to 29.9 ± 0.8 mm, respectively [11].

However, some differences between our results and previously published data were observed. In the study conducted by Borugă et al. [17], the same reference strain of *P. aeruginosa* ATCC 27853 was susceptible to the *T. vulgaris* essential oil, with inhibition zones ranging from 11.82 ± 0.27 to 14.13 ± 0.19 mm (moderate activity), whereas no inhibition of this strain was detected in our experiment. Borugă et al. [17] also demonstrated pronounced activity against *K. pneumoniae* ATCC 13882 and *C. albicans* ATCC 10231. In their study, the inhibition zones ranged from 30.21 ± 0.12 to 33.93 ± 0.14 mm and from 15.14 ± 0.38 to 30.20 ± 0.17 mm for *K. pneumoniae* and *C. albicans*, respectively. In contrast, our sample did not inhibit *K. pneumoniae* No. 43 (a clinical strain). It should be noted, however, that different strains of *K. pneumoniae* were used in the two studies. However, our results and the findings revealed by Borugă et al. [17] showed that the essential oil of the thymol chemotype has strong activity for *S. aureus* and *E. coli*.

Overall, the results indicate that the investigated thymol-rich *T. vulgaris* essential oil exhibited selective antimicrobial activity, with the strongest effect against *S. aureus* ATCC 25923, followed by *E. coli* ATCC 25922 and *S. epidermidis* ATCC 12228. The high thymol content (49.9%), together with γ-terpinene (11.2%), *p*-cymene (7.9%), carvacrol (4.5%), and other constituents, may contribute to the observed antibacterial activity. At the same time, differences from literature data, particularly regarding *P. aeruginosa* and *K. pneumoniae*, indicate that antimicrobial activity cannot be predicted solely from the concentration of the major constituent and is likely determined by the complete chemical profile of the essential oil, possible synergistic interactions among its constituents, microbial strain susceptibility, and the conditions of the antimicrobial assay.

The key constituents in the essential oil of *O. basilicum* (Ob17-23) were linalool (38.7%), neral (9.4%), nerol (4.0%), and terpinen-4-ol (2.4%). It seems that this sample of the plant belongs to the linalool chemotype. Our studies are in line with the studies of Dris et al. [42]. The principal components of the essential oil of *O. basilicum* collected in northeast of Albania were linalool (22.5%), linalyl acetate (53.9%), α-terpineol (4.6%), and eucalyptol (3.4%) [42].

According to Dris et al. [42] and Joshi [18], there are four chemotypes of *O. basilicum* (linalool, methyl cinnamate, methyl chavicol, and eugenol). Linalool or a mixture of linalool and methyl chavicol are the principal components of the European chemotype, which grows in Bulgaria, France, Italy, Egypt, and South Africa.

In the essential oil of *O. basilicum* grown at the attitude of 800 m in the district of Belgaum (N 15.88668; E 74.52353) (Karnataka, India), methyl eugenol (39.3%) and methyl chavicol (38.3%) were identified as the main components. This essential oil was active against Gram-positive (*S. faecalis* (NCIM 2080), *S aureus* (NCIM 2079), *S. epidermidis* (NCIM 2493), *Micrococcus flavus* (NCIM 2379), *M. luteus* (NCIM 2103), *B. subtilis* (NCIM 2063)), Gram-negative bacteria (*E. coli* (NCIM 2574), *Enterobacter aerogenes* (NCIM 2694), *K. pneumoniae* (NCIM 2957), *P. aeruginosa* (NCIM5029), *Proteus vulgaris* (NCIM 2813), *P. mirabilis* (NCIM 2241), *Serratia marcescens* (NCIM 2078), *Salmonella typhimurium* (NCIM 2501), and fungi with minimal bactericidal concentrations (MBC) in the range from 0.143 ± 0.031 to 0.572 ± 0.127 mg/mL, from 0.781 ± 0.382 to 1.875 ± 0.684 mg/mL, and antifungal concentrations from 0.312 ± 0.171 to 0.442 ± 0.207 mg/mL, respectively [18]. This Indian chemotype can belong to the mixed methyl eugenol-methyl chavicol chemotype.

The essential oil of *O. basilicum* of Turkish origin contained methyl eugenol (78.0%), α-cubebene (6.2%), nerol (0.8%), and ε-muurolene (0.7%) [29]. It seems that the Turkish sample belongs to the methyl eugenol chemotype. According to Dris et al. [42] and Joshi [18], the tropical chemotype is rich in methyl cinnamate. It grows in India, Pakistan, and Guatemala. The chemotype of Reunion is rich in methyl chavicol, which grows in Madagascar, Thailand and Vietnam, whereas the eugenol chemotype is characteristic for Northern Africa, Eastern Europe, and Northern Asia [18,42].

As it was summarized recently, the active constituents of the *Hyssopus officinalis* essential oil include bicyclic monoterpenes (β-pinene, *cis*-pinocamphone, pinocamphone, *L*-pinocamphene, pinocarvone) and other terpenoids depending on chemotype and locality [34,35,36]. Our sample of the essential oil of *H. officinalis* contained 51.3% of 3-pinanone, 18.6% of pinocarvone, and 9.1% β-pinene. By and large, these substances are described as components of the *H. officinalis* essential oil [34].

Hyssop essential oil is a clear, colorless or pale yellowish-to-green liquid with a camphor-like aroma, warm and spicy notes, and a pungent taste [36]. As stated by Atazhanova et al. [35], such components are characteristic for this species: pinocarvone (5.3–36.3%), pinocamphone and *cis*-pinocamphone (16.4–61.6%), α-pinene, β-pinene, sabinene, myrcene, phellandrene, linalool, myrtenol, elemol, and germacrene-D. The relative content of these two ketones, pinocamphone and *cis*-pinocamphone, ranges from 2.9 to 63.6%. Moreover, they are in dynamic equilibrium [35].

The presence of numerous subspecies, cultivars, and chemotypes within this species somewhat diversifies its chemical composition and potential biological activity [35]. After analyzing the original articles on the chemical composition of the essential oil of *H. officinalis* collected from various regions, Nurzyńska-Wierdak [36] has made the following generalizations: the major components of the essential oil include *cis*-pinocamphone (4.68–65.40%), sabinene (28.4–57.2%), *trans*-pinocamphone (1.30–53.0%), heneikozene (9.49–37.0%), pinocarvone (0.44–29.17), geranyl acetate (7.5–25.0%) camphor (0.8–23.60%), and *β*-pinene (6.30–21.90%). The changes in the volatile oil content and composition were observed during ontogeny and also depended on genetic and environmental factors [36].

The composition of the essential oils from wild-growing *H. officinalis* plants in Montenegro exhibited significant variability [43]. Three distinct chromatographic profiles of these essential oils were identified: (1) the essential oils that are rich in 1,8-cineole and moderately rich in β-pinene, but low in *cis*-pinocamphone; (2) the essential oils that are abundant in β-pinene, limonene, *cis*-pinocamphone, and methyl eugenol, yet relatively low in 1,8-cineole; and (3) the essential oils that are relatively rich in 1,8-cineole, limonene, β-pinene, and *cis*-pinocamphone. These essential oils showed a significant reduction in DNA damage when tested in vitro [43].

All the tested essential oils showed strong antimicrobial activity in the disk diffusion method. The growth inhibition zones of *E. coli* were larger compared to the standard antimicrobial drug substances, ciprofloxacin (16 mm), with the exception of the sample of Ho20 (11 mm). The largest growth inhibition zones were observed for the essential oil of *M. didyma* (Md3-1) and *S. coerulea* (Sc19). In the case of the Gram-positive bacteria, *S. aureus* and *S. epidermidis*, growth inhibition zones (18–32 mm) were larger as well compared to ciprofloxacin (16 and 18 mm, respectively), with the exception of Sh14 (14 mm for *S. epidermidis*). The largest growth inhibition zones were observed for the essential oil of *S. coerulea* (Sc19): 32 and 29 mm for *S. aureus* and *S. epidermidis*, respectively.

Despite some merits of our studies (simultaneous evaluation of eight cultivars grown on the same plots in a new region), there is one drawback. We could not measure the values of MIC because of the small amounts of the essential oils. However, the disk diffusion method showed some perspectives of the developed cultivars. Moreover, our studies show that all the chemotypes will be interesting from an economic point of view for the development of pharmaceutical and cosmetic products.

## 3. Materials and Methods

### 3.1. Chemicals

Standard compounds used for GC analyses and biological testing were obtained from Sigma-Aldrich (St. Louis, MO, USA); the compounds included thymol and carvacrol.

### 3.2. Plants Cultivation

The plant cultivars of five genera, *Thymus*, *Monarda*, *Ocimum*, *Hyssopus*, and *Satureja*, were cultivated in central Ukraine in 2023. In the autumn of 2022, a duplicate collection of aromatic and medicinal plants was laid firstly in the conditions of the central forest-steppe. The research was conducted in 2023 in the conditions of the central forest-steppe of Ukraine in the Lysyansky district of the Cherkasy region. The district is located on the Dnipro Upland of the East European Plain, in the basin of the Gnyly Tikich River.

The climate of the central forest-steppe is temperate continental, with relatively mild, little-snowy winters and warm, moderately humid summers. The average temperature in June was 20 °C with variations from 14.6 °C to 25.0 °C, whereas the average temperature in July was 21.1 °C with variations from 17.8 °C to 24.8 °C. The precipitation was 22.3 mm and 149.7 mm, respectively, in June and July 2023. The average temperature, variations in a temperature and precipitation in this region are provided on the official site [44]. The soil of the area where the plants grew is a typical chernozem.

### 3.3. Morphological Characteristics

Some morphological characteristics (height and diameter of the plants, number of shoots, width and length of leaves, number of flowers in rings, color of leaves and flowers, mass of the plants) were evaluated in the flowering stage. These measurements were carried out on 5–10 samples. Each cultivar was grown on the plot of 6.75 m^2^.

### 3.4. Essential Oil Distillation

All the plants were collected in the summer of 2023 and cut into small parts (2.0–2.5 cm). All the essential oils were obtained from the fresh-cut aerial parts of the plants via hydrodistillation for 45–60 min using a Clevenger-type apparatus (about 100 g for each repetition) according to the procedure described in [18,29,42] with slight modifications. The obtained essential oils were further kept in the fridge at 4 °C before analysis by GC-MS and antibacterial activity. The yield of the obtained essential oils was calculated based on a fresh mass and dried mass of the herbal substance for three repetitions. For the calculations of yields in reference to dry herbal material, this material was dried at a temperature of 45 °C to a constant mass.

### 3.5. GC-MS Analysis

GC-MS analysis was performed using a Shimadzu GC/MS-QP2010 Ultra system equipped (Shimadzu Corporation, Kyoto, Japan) with an electron ionisation (EI) source and a split/splitless injector. Chromatographic separation was achieved on an Rxi-5 ms capillary column (30 m × 0.25 mm, 0.25 μm film thickness; 5% diphenyl/95% dimethylpolysiloxane). Helium (99.999%) was used as the carrier gas at a constant flow rate of 1.22 mL/min.

The oven temperature program was set to 50 °C (2 min), increased to 210 °C at 3 °C/min, then to 310 °C at 40 °C/min, and held for 5 min. The injector temperature was maintained at 250 °C, and the injection volume was 1 μL. The split ratio was 1:50. The MS interface and ion source temperatures were set at 280 °C and 200 °C, respectively. The mass spectrometer was operated in positive EI mode at 70 eV. Full-scan acquisition (*m*/*z* 35–500) was used for compound identification, while quantitative analysis was performed in selected ion monitoring (SIM) mode. Data acquisition and processing were carried out using LabSolutions GC/MS software (Version 5.71). Identification of volatile compounds was carried out using mass spectra library search (NIST 14, WR10, WR10R) and compared with the acquired MS data of the standards and mass spectral data from the literature.

### 3.6. Antibacterial Activity

The investigated samples of the essential oils were evaluated for antibacterial activity using the agar diffusion method [45,46].

The following reference and clinical bacterial strains were used in this study: the Gram-positive bacteria *Staphylococcus epidermidis* ATCC 12228, *Staphylococcus aureus* ATCC 25923; and the Gram-negative bacteria *Pseudomonas aeruginosa* ATCC 27853, *Klebsiella pneumoniae* clinical isolate No. 43, and *Escherichia coli* ATCC 25922. The bacterial strains were cultured on Mueller–Hinton agar. For each test strain, a microbial suspension equivalent to 0.5 McFarland standard (approximately 1.5 × 10^8^ CFU/mL) was prepared according to the standard procedure for antimicrobial susceptibility testing. The suspension was uniformly inoculated onto the agar surface. The sterile paper disks were impregnated with the tested samples (20 µL) and placed on the inoculated agar plates. The Petri dishes were incubated at 37 °C for 24 h. The diameter of the growth inhibition zone around each disk was measured in millimeters. Ciprofloxacin was used as the positive control for bacterial strains. All the experiments were performed in triplicate (n = 3).

In our studies, we used the classification proposed by Chen et al. [6]. The essential oil antibacterial activity was classified into three groups: strong activity (inhibition zone diameter > 20 mm), moderate activity (12 < inhibition zone diameter < 20 mm), and weak activity (inhibition zone diameter < 12 mm) [6].

## 4. Conclusions

In the present study, we tested eight cultivars from five Lamiaceae genera (*Monarda*, *Satureja*, *Thymus*, *Ocimum*, and *Hyssopus*) for the key morphological features, chemical composition of the essential oils by GC-MS and antibacterial activity. In six cultivars, carvacrol, thymol, *γ*-terpinene, and cymene were the main components. Moreover, *M. bradburiana*, one *M. didyma* cultivar (Md4-2), and *S. hortensis* belonged to the carvacrol chemotype, whereas *M. didyma* (Md3-1) and *T. vulgaris* belonged to the thymol chemotype of the respective species. All the tested essential oils showed strong antimicrobial activity in the disk diffusion method. In the case of the Gram-positive bacteria, *S. aureus* and *S. epidermidis*, the growth inhibition zones were larger as well compared to ciprofloxacin, with the exception of *S. hortensis* (Sh14). The largest growth inhibition zones were observed for the essential oil of *S. coerulea* for *S. aureus* and *S. epidermidis*, respectively. The growth inhibition zones of *E. coli* were larger compared to those of the standard antimicrobial drug substance, ciprofloxacin, with the exception of the sample Ho20. The largest growth inhibition zones were observed for the essential oil of *M. didyma* (Md3-1) and *S. coerulea*.

In summary, our studies describe some newly developed cultivars (*Monarda bradburiana*, *Monarda didyma*, *Satureja hortensis*, *Satureja coerulea*, *Thymus vulgaris*, *Ocimum basilicum,* and *Hyssopus officinalis*). These studies have widened the knowledge about their morphological features, as well as the chemical profiles and antibacterial activity of their essential oils. The microbiological studies demonstrated that the studied essential oils are likely to induce antibacterial activity due to their specific chemical composition. By and large, the morphological studies of the plants, and chemical composition and antibacterial activity of the essential oils highlight the need to continue further studies to make a judgment about the repeatability of results for these cultivars cultivated across other years on the same plots.

## Figures and Tables

**Table 1:** Features of the tested plants.

№	Plant	Code	Flowering in 2023			Average Yield, g/Plant	Yield EO, %Dried/%Fresh	Date of Distillation
			Start	Mass	End			
1	*Monarda didyma*	Md3-1	25.06	9.07	20.07	139	2.10 ± 0.21/ 0.64 ± 0.064	20.07
2	*Monarda didyma*	Md4-2	27.06	15.07	26.07	162	0.99 ± 0.14/ 0.30 ± 0.04	20.07
3	*Monarda bradburiana*	Mb5-09	29.06	17.07	27.07	115	1.65 ± 0.085/ 0.50 ± 0.025	21.07
4	*Satureja hortensis*	Sh14-22	17.07	25.07	8.08	96.6	2.40 ± 0.105/ 0.70 ± 0.03	29.07
5	*Thymus vulgaris*	Tv16-22	18.05	26.05	15.06	42	1.60 ± 0.125/ 0.48 ± 0.037	20.08
6	*Ocimum basilicum*	Ob17-23	20.07	7.08	1.09	85.5	0.32 ± 0.046/ 0.07 ± 0.01	20.08
7	*Satureja coerulea*	Sc19-23	26.08	31.08	11.09	80	1.22 ± 0.10/ 0.32 ± 0.025	27.08
8	*Hyssopus officinalis*	Ho20-7-18	10.08	20.08	15.09	110	0.70 ± 0.07/ 0.21 ± 0.02	27.08

**Table 2:** The chemical composition of the studied essential oils determined by GC-MS.

Normalized Name	R Time	RI	*Hyssopus officinalis*	*Monarda bradburiana*	*Monarda didyma 3-1*	*Monarda didyma 4-2*	*Ocimum basilicum*	*Satureja hortensis*	*Satureja coerulea*	*Thymus vulgaris*
*Monoterpene hydrocarbons*			**15.54**	**18.68**	**24.15**	**15.25**	**-**	**25.69**	**25.06**	**22.59**
α-Thujene	7.635	951	0.23	1.06	1.54	0.73	-	0.62	1.39	0.58
α-Pinene	7.869	958	-	0.32	0.36	-	-	-	0.48	-
2-Pinene	8.401	973	0.54	-	-	0.17	-	0.31	-	0.32
Camphene	8.408	973	0.12	0.05	0.06	-	-	-	0.09	0.22
4(10)-Thujene (sabinene)	9.385	1001	1.96	-	7.02	4.36	-	0.11	0.18	0.09
β-Pinene	9.489	1003	9.12	0.10	0.14	0.08	-	0.15	0.19	0.13
β-Myrcene	10.123	1018	1.36	1.29	2.07	1.12	-	1.18	1.95	0.97
α-Phellandrene	10.627	1030	-	0.21	0.25	-	-	0.08	0.26	0.08
1,3,6-Octatriene, 3,7-dimethyl-, (Z)- (cis-beta-Ocimene)	10.863	1036	-	-	-	-	-	-	-	0.05
3-Carene	10.873	1036	-	0.10	0.10	-	-	-	0.06	-
α-Terpinene	11.149	1043	-	1.48	1.93	0.52	-	0.66	-	0.72
2-Carene	11.149	1043	-	-	-	-	-	-	2.32	-
*p*-Cymene	11.483	1051	0.25	6.34	5.78	4.26	-	6.23	3.90	7.92
D-Limonene	11.663	1055	-	0.59	-	-	-	-	-	-
Cyclohexene, 1-methyl-5-(1-methylethenyl)-, (R)-	11.664	1055	1.36	-	-	-	-	0.32	0.50	0.30
Limonene	11.665	1055	-	-	0.83	0.42	-	-	-	-
trans-β-Ocimene	12.126	1066	-	-	-	-	-	-	0.07	-
β-Ocimene	12.569	1077	0.60	-	-	-	-	-	0.11	-
γ-Terpinene	13.007	1087	-	7.14	4.07	3.59	-	16.03	13.56	11.21
*Oxygenated monoterpenes*			**78.21**	**67.76**	**65.30**	**76.89**	**78.64**	**69.07**	**66.26**	**69.01**
1,8-Cineole (Eucalyptol)	11.805	1059	0.21	-	0.27	0.41	0.42	-	0.41	0.87
4-Thujanol	13.710	1104	1.25	1.92	1.62	2.65	0.61	0.26	1.48	2.75
trans-Linalool oxide (furanoid)	14.000	1110	-	-	-	-	1.76	-	-	-
4-Thujanol, cis-(±)-	14.776	1127	-	-	-	-	0.46	0.21	-	0.45
Linalool	14.867	1130	0.67	0.41	-	0.48	38.65	-	1.01	4.70
Thujone	15.573	1145	0.29	-	-	-	-	-	-	-
Isopinocarveol	16.590	1168	0.08	-	-	-	-	-	-	-
(+)-2-Bornanone	16.819	1173	-	-	-	-	-	-	-	0.44
trans-Pinocamphone	17.584	1190	-	-	-	-	-	-	0.32	-
3-Pinanone	17.653	1192	51.29	-	-	-	-	-	-	-
Pinocarvone	17.735	1194	18.45	-	-	-	-	-	-	-
endo-Borneol	17.831	1196	0.05	-	-	0.13	-	0.08	0.29	1.73
7-Oxabicyclo[4.1.0]heptane, 1-methyl-4-(1-methylethenyl)-	17.925	1198	-	-	-	-	-	0.49	-	-
2,6-Dimethyl-3,5,7-octatriene-2-ol, E,E-	17.937	1198	-	-	-	-	-	-	-	0.40
*cis*-Pinocamphone	18.201	1204	1.13	-	-	-	-	-	-	-
Terpinen-4-ol	18.378	1208	0.37	0.46	0.50	0.95	2.38	0.20	0.53	0.51
Isogeranial	18.686	1215	-	-	-	-	0.27	-	-	-
α-Terpineol	19.024	1222	0.28	0.15	0.23	0.34	-	0.13	0.20	0.31
Bicyclo[3.1.1]hept-2-ene-2-methanol, 6,6-dimethyl-	19.258	1228	3.37	-	-	-	-	-	-	-
2,6-Octadien-1-ol, 3,7-dimethyl-, (Z)-	20.749	1261	-	-	-	-	4.72	-	-	-
Benzene, 2-methoxy-4-methyl-1-(1-methylethyl)- 2	21.048	1268	-	-	-	-	-	-	-	0.06
Neral	21.308	1274	-	-	-	-	9.42	-	-	-
2,6-Octadienal, 3,7-dimethyl-, (Z)-	21.381	1276	-	-	-	-	-	-	-	0.12
Benzene, 2-methoxy-1-methyl-4-(1-methylethyl)- 2	21.460	1277	-	-	-	-	-	-	-	0.17
Benzene, 2-methoxy-1-methyl-4-(1-methylethyl)-	21.464	1278	-	-	-	-	-	0.15	-	-
(-)-Carvone	21.491	1278	-	-	-	-	-	-	0.09	-
Thymoquinone	21.701	1283	-	0.17	-	-	-	-	-	-
Nerol	21.956	1289	-	-	-	-	3.98	-	-	-
Geraniol	22.038	1290	-	0.12	-	-	-	-	-	0.18
Citral	22.704	1306	-	-	-	-	12.60	-	-	0.13
Phenol, 5-methyl-2-(1-methylethyl)-	23.392	1322	-	-	-	-	-	0.12	-	-
Bornyl acetate	23.432	1323	-	-	-	-	-	-	-	0.70
Thymol	23.685	1329	0.05	0.72	58.48	1.65	1.02	0.54	12.42	49.89
Myrtenate methyl	23.840	1332	0.39	-	-	-	-	-	-	-
Phenol, 2-Methyl-5-(1-Methylethyl) 2-	23.845	1332	-	-	-	-	-	-	-	0.26
Carvacrol	24.118	1339	0.25	63.58	4.20	69.91	0.90	66.32	49.35	4.53
Isoascaridole	24.327	1344	-	-	-	0.09	-	-	-	0.08
6-Octen-1-ol, 2,3-epoxy-3,7-dimethyl-	25.067	1361	-	-	-	-	0.18	-	-	-
Myrtenyl acetate	25.085	1361	0.08	-	-	-	-	-	-	-
Oxiranemethanol, 3-methyl-3-(4-methyl-3-pentenyl)-	25.524	1372	-	-	-	-	0.42	-	-	-
Geranic acid	26.286	1389	-	-	-	-	0.14	-	-	-
Phenol, 5-methyl-2-(1-methylethyl)-, acetate	26.336	1391	-	-	-	-	-	-	-	0.17
(2Z)-3,7-Dimethyl-2,6-Octadienyl Acetate	26.813	1402	-	-	-	-	0.50	-	-	-
Carvacrol acetate	27.132	1410	-	0.05	-	0.28	-	0.18	0.16	-
Neryl acetate	27.638	1422	-	-	-	-	0.21	-	-	-
Geranyl acetate	27.639	1422	-	0.18	-	-	-	-	-	0.07
(E)-3,7-Dimethylocta-2,6-dien-1-yl 3-methylbutanoate	31.402	1515	-	-	-	-	-	-	-	0.45
Geranyl butyrate	34.810	1604	-	-	-	-	-	-	-	0.04
3-Methyl-2-(2-methyl-2-butenyl)-furan	52.193	2126	-	-	-	-	-	0.39	-	-
*Sesquiterpene hydrocarbons*			**1.67**	**0.78**	**0.96**	**0.41**	**1.26**	**0.58**	**3.50**	**0.89**
(-)-β -Bourbonene	27.621	1422	0.18	-	-	-	-	-	-	-
α-Gurjunene	28.672	1447	0.08	-	-	-	-	-	-	-
β-Caryophyllene	29.058	1457	0.28	0.26	0.28	0.34	0.38	-	1.96	0.89
cis-α-Bergamotene	29.782	1474	-	-	-	-	0.40	-	-	-
Humulene	30.474	1491	-	-	-	-	-	-	0.09	-
Caryophyllene <9-epi-(E)->	30.773	1498	0.30	-	-	-	-	-	-	-
γ-Muurolene	31.445	1516	-	0.10	-	-	-	-	-	-
Germacrene D	31.608	1520	0.69	0.20	0.48	0.07	-	-	0.56	-
trans-β-Bergamotene	31.778	1525	-	-	0.20	-	-	-	-	-
α-Guaiene	32.181	1535	-	0.09	-	-	-	-	-	-
Bicyclogermacrene	32.248	1537	0.14	-	-	-	-	-	0.21	-
β -Bisabolene	32.758	1550	-	-	-	-	-	0.58	0.62	-
delta-Cadinene	33.336	1565	-	0.13	-	-	-	-	0.06	-
α-Z-Bisabolene	34.099	1585	-	-	-	-	0.48	-	-	-
*Oxygenated sesquiterpenes*			**0.93**	**-**	**-**	**0.29**	**0.55**	**0.86**	**0.51**	**2.09**
Z-Nerolidyl acetate	32.959	1555	-	-	-	-	-	-	-	0.13
(-)-Spathulenol	35.402	1620	0.55	-	-	-	-	0.43	0.21	-
Caryophyllene oxide	35.610	1625	0.14	-	-	0.22	0.55	0.43	0.24	1.14
Ledol	36.394	1647	0.09	-	-	-	-	-	-	-
2-((2S,4aR)-4a,8-Dimethyl-1,2,3,4,4a,5,6,7-octahydronaphthalen-2-yl)propan-2-ol	37.011	1663	-	-	-	-	-	-	-	0.24
tau-Cadinol	37.800	1685	0.15	-	-	-	-	-	-	0.34
epi-β-Cadinol	37.814	1685	-	-	-	-	-	-	0.06	-
(1R,7S,E)-7-Isopropyl-4,10-dimethylenecyclodec-5-enol	39.459	1732	-	-	-	0.07	-	-	-	-
6-Isopropenyl-4,8a-dimethyl-1,2,3,5,6,7,8,8a-octahydro-naphthalen-2-ol	39.463	1732	-	-	-	-	-	-	-	0.06
Nerolidol-epoxyacetate	40.218	1753	-	-	-	-	-	-	-	0.06
Oplopanone	41.308	1784	-	-	-	-	-	-	-	0.12
*Diterpene hydrocarbons*			**-**	**-**	**-**	**0.12**	**-**	**-**	**-**	**-**
1,3,6,10-Cyclotetradecatetraene, 3,7,11-trimethyl-14-(1-methylethyl)-, [S-(E,Z,E,E)]-	35.000	1609	-	-	-	0.12	-	-	-	-
*Other oxygenated terpenoids*			**-**	**-**	**-**	**0.26**	**-**	**-**	**-**	**-**
6-Methyl-γ-Ionone	50.931	2084	-	-	-	0.26	-	-	-	-
*Phenylpropanoids*			**0.50**	**0.10**	**-**	**0.24**	**-**	**-**	**-**	**0.13**
Anisole, p-allyl-	19.337	1230	-	-	-	-	-	-	-	0.13
Estragole	19.348	1230	0.32	0.10	-	0.24	-	-	-	-
Methyleugenol	28.505	1443	0.18	-	-	-	-	-	-	-
*Aliphatic hydrocarbons*			**2.82**	**2.29**	**2.15**	**0.94**	**8.14**	**2.28**	**2.36**	**2.86**
Cyclohexane	2.192	–	2.78	2.24	2.07	-	1.28	2.23	2.33	2.82
Heptane	2.486	716	0.04	0.05	0.08	-	0.30	0.05	0.03	0.04
Naphthalene, 1,2,3,4,4a,5,6,7-octahydro-4a-methyl-	46.282	1934	-	-	-	0.94	-	-	-	-
Octadecane	56.120	2259	-	-	-	-	0.62	-	-	-
Eicosane	58.877	2414	-	-	-	-	3.64	-	-	-
Nonacosane	59.839	–	-	-	-	-	2.30	-	-	-
*Other non-terpenoid oxygenated compounds*			**0.02**	**9.20**	**7.06**	**5.19**	**0.79**	**0.16**	**1.76**	**2.09**
Butyl alcohol-1-d1	2.031	–	-	-	1.23	-	-	-	-	-
Methyl 2,5,8,11,14,17-hexaoxanonadecan-19-oate	2.992	757	0.02	-	-	-	-	-	-	-
2-methyl-butanoic acid methyl ester	3.596	803	-	-	-	-	-	-	-	0.21
(3Z)-3-Hexen-1-Ol	5.411	881	-	-	-	-	-	-	-	0.06
3-Hexen-1-ol, (Z)-	5.411	881	-	-	-	-	-	-	0.04	-
7-Octen-4-ol	9.646	1007	-	-	4.68	4.78	-	0.16	1.61	1.63
1-Octen-3-ol	9.648	1007	-	7.73	-	-	-	-	-	-
3-Octanone	9.964	1015	-	0.13	0.51	0.11	-	-	-	0.07
3-Octanol	10.339	1024	-	0.31	0.64	0.11	-	-	0.05	0.12
1-Octanol	13.608	1101	-	0.11	-	-	-	-	-	-
1-Nonen-3-ol	14.765	1127	-	0.18	-	0.10	-	-	0.06	-
Nonanal	15.076	1134	-	0.26	-	0.09	-	-	-	-
Acetic acid, octyl ester	20.076	1246	-	-	-	-	0.12	-	-	-
2,7-Dimethyl-2,7-octanediol	25.672	1375	-	-	-	-	0.37	-	-	-
Propanoic acid, 2-methyl-, 3-hexenyl ester, (Z)-	26.523	1395	-	-	-	-	0.30	-	-	-
n-Nonadecanol-1	52.633	2141	-	0.48	-	-	-	-	-	-
*Nitrogen-containing compounds*			**0.33**	**0.77**	**-**	**-**	**4.34**	**0.33**	**0.55**	**-**
Hydrazine, (1,1-dimethylethyl)-	2.027	–	0.33	-	-	-	-	-	0.55	-
Acetic acid, hydrazide	2.027	–	-	-	-	-	4.34	-	-	-
Valeric acid hydrazide	2.028	–	-	0.77	-	-	-	-	-	-
Benzo[g]pteridine-2,4(3H,10H)-dione, 8-hydroxy-7,10-dimethyl-	2.028	–	-	-	-	-	-	0.33	-	-
*Other/Unclassified compounds*			**-**	**0.06**	**-**	**-**	**-**	**0.43**	**-**	**-**
1-Hexanone, 1-[4-(4-methyl-3-pentenyl)-3-cyclohexen-1-yl]-	50.098	2056	-	0.06	-	-	-	-	-	-
Total identified constituents			**100.02**	**99.64**	**99.62**	**99.59**	**93.72**	**98.97**	**100.00**	**99.66**

**Table 3:** Results of antimicrobial activity of the tested essential oils.

Microorganisms		Zone of Growth Inhibition (mm ± SE), n = 3								
		Md 3-1	Md 4-2	Mb 5-09	Sh 14-22	Tv 16-22	Ob 17-23	Sc 19-23	Ho20-7-18	Ciprofloxacin (Control)
1	*Staphylococcus aureus* ATCC 25923	26.3 ± 0.2	20 ± 0.3	23 ± 0.2	22.2 ± 0.3	25 ± 0.2	23 ± 0.3	32 ± 0.6	23 ± 0.3	16 ± 0.3
2	*Staphylococcus epidermidis* ATCC 12228	20 ± 0.3	ND	ND	14 ± 0.2	20 ± 0.3	18 ± 0.3	29 ± 0.3	25 ± 0.2	18 ± 0.3
3	*Escherichia coli* ATCC 25922	32 ± 0.3	ND	26 ± 0.2	24 ± 0.3	22 ± 0.3	20 ± 0.6	31 ± 0.2	11 ± 0.3	16 ± 0.2
4	*Pseudomonas aeruginosa* ATCC 27853	19 ± 0.3	6	ND	11 ± 0.4	6	10 ± 0.3	20 ± 0.3	22 ± 0.3	14 ± 0.3
5	*Klebsiella pneumoniae* No 43	-	6	20 ± 0.3	24 ± 0.4	6	10 ± 0.3	32 ± 0.2	16 ± 0.3	20 ± 0.3

Abbreviations: The diameters of the growth inhibition zones including the diameter of the filter paper disks, 6 mm, were measured in millimeters; ND—requires repeated testing; 6 mm—no inhibition zone observed around the disks with a diameter of 6 mm; - not tested.

## Data Availability

Data will be made available on request.
